# Genetic Evidence Supporting a Role for Brain Region Volume and Functional Network Alterations in Major Depression

**DOI:** 10.1002/advs.202506032

**Published:** 2025-07-11

**Authors:** Ming‐Min Xu, Nan Li, Yi‐Wen Lv, Wen‐Miao Yang, Zhi‐Yun Yu, Yi‐Min Zhang, Yu Guo, Xiao‐Yin Chen

**Affiliations:** ^1^ School of Traditional Chinese Medicine Jinan University Guangzhou 510632 China; ^2^ Guangzhou Key Laboratory of Formula‐Pattern of Traditional Chinese Medicine (Jinan University) Guangzhou 510632 China

**Keywords:** brain functional network, brain region volume, daily behavioral prevention, major depression, Mendelian randomization, risk prediction

## Abstract

Despite emerging evidence of altered brain region volumes and functional networks in major depression (MD), causal relationships remain unexplored. Here, using genetic variants related to human brain morphometry (*n* = 36778) and functional networks (*n* = 47276), bidirectional two‐sample and multivariable Mendelian randomization (MR) is performed to systematically investigate causal relationships between 83 brain‐wide volumes, 191 resting‐state functional magnetic resonance imaging phenotypes, and MD to identify core brain region volumes and functional networks associated with MD onset. Unlike other severe psychiatric disorders, reductions in left and right ventral diencephalon and thalamus volumes, together with decreased functional connectivity between the precuneus, cuneus, or cingulate gyri and frontal regions within the triple network (default mode network, central executive network, and salience network) uniquely predict MD onset. Using the identified markers as mediators in two‐step MR recommends quitting smoking, moderately increasing alcohol frequency without developing alcohol use disorder, engaging in walking for leisure, and participating in exercises (e.g., swimming, cycling, keep fit, bowling) to positively influence imaging phenotypes and reduce MD risk. These findings enhance the understanding of predictive outcomes in brain imaging and support proactive prevention of severe mental health disorders through behavioral modifications.

## Graphical abstract

Genome‐wide analyses indicate that reduced ventral diencephalon and thalamus volumes, along with decreased functional connectivity within the triple network (DMN/CEN/SN), are uniquely associated with the risk of major depression (MD) onset, compared to other similar severe psychiatric disorders. Mediation modeling further suggests that smoking cessation, moderate alcohol intake, leisure walking, and participating in regular exercises may help prevent MD.

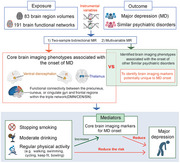

## 1 Introduction

Major depression (MD) is a debilitating mental health condition marked by severe and persistent symptoms such as profound sadness, an inability to derive pleasure from previously enjoyable activities, and significant impairment in daily functioning.^[^ ^1^, ^2^ ^]^ Unlike in mild or moderate depression, individuals with MD experience a profound deterioration in their quality of life.^[^ ^1^, ^2^ ^]^ MD is also associated with an increased risk of developing co‐morbid physical disease^[^ ^3^ ^]^ and suicide,^[^ ^4^ ^]^ incurring a considerable personal, social, and economic burden. Epidemiological data indicate that ≈3.8% of the global population suffers from MD, with significant demographic heterogeneity.^[^ ^5^ ^]^ From 1990 to 2019, the global incidence of MD, prevalence rates, and disability‐adjusted life years (DALYs) increased by 59.1%, 59.6%, and 58.6%, respectively.^[^ ^6^ ^]^ An international analysis of 156 331 respondents reported a lifetime prevalence of any mental disorder at 28.6% for men and 29.8% for women, with MD among the most prevalent reported disorders.^[^ ^7^ ^]^ Consequently, MD has become a leading global public health challenge.

MD arises through a complex interplay between genetic predisposition, neurobiological alterations, psychological factors, and environmental stressors. This multifaceted and multifactorial etiology contributes to treatment resistance, making effective management challenging.^[^ ^1^, ^8^ ^]^ Given the severity of MD, there is an urgent need for precise and timely prediction and prevention strategies to stop progression from mild or moderate depressive disorder to MD. Early detection and subsequent intervention based on individual behaviors may significantly reduce the incidence of severe depression, improve patient outcomes, and reduce healthcare costs. However, MD shares notable clinical similarities with other common and severe psychiatric disorders including bipolar disorder,^[^ ^9^, ^10^ ^]^ schizophrenia,^[^ ^10^ ^]^ schizotypal,^[^ ^11^ ^]^ and delusional disorders,^[^ ^12^ ^]^ as well as autism spectrum disorder (ASD),^[^ ^13^ ^]^ particularly in terms of mood disturbances, cognitive impairments, and social withdrawal. These similarities complicate the differential diagnosis of MD and challenge accurate prediction.^[^ ^14^, ^15^ ^]^

There is emerging clinical evidence of significant alterations in brain region volumes and functional networks associated with MD and other severe psychiatric conditions.^[^ ^16^, ^17^, ^18^, ^19^, ^20^, ^21^, ^22^ ^]^ However, causal relationships between these brain imaging phenotypes and MD onset remain largely unexplored. Conventional cohort studies are often costly and time‐consuming, so few have been performed and the findings are often inconsistent. In addition, observational studies are, by design, often weakened by confounding and the reverse influence of MD, making it difficult to establish clear causal links. Moreover, there may be significant overlap in brain region volumes and functional networks between MD and other similar severe psychiatric disorders, complicating the differentiation of MD onset using this approach. Therefore, there is a pressing need for further research to elucidate these relationships, ultimately enhancing our understanding and informing more effective predictive and preventive strategies for the daily management of MD.

Mendelian randomization (MR) is an alternative epidemiological method for modeling and inferring the potential causality of an exposure on an outcome by utilizing genetic variants, typically single nucleotide polymorphisms (SNPs), as instrumental variables (IVs).^[^ ^23^ ^]^ As genetic variants are randomly allocated during meiosis and fertilization, they are relatively independent of self‐selected behaviors and are established well before disease onset, thereby minimizing the influence of common factors that confound exposure–outcome associations in conventional observational analyses.^[^ ^23^, ^24^ ^]^ In addition, since an individual's genotype is determined at conception and cannot be modified by subsequent disease outcomes, the direction of causation will always be from the genetic variant to the trait of interest, eliminating the potential for reverse causation.^[^ ^23^, ^24^ ^]^ Therefore, estimates from MR can provide more reliable insights into causal relationships between risk factors and disease outcomes. Over recent years, several MR studies have provided evidence for a causal association between brain imaging phenotypes and the risk of MD.^[^ ^25^, ^26^, ^27^, ^28^, ^29^, ^30^ ^]^ However, these investigations have failed to identify specific predictive core brain regions and functional networks associated with MD onset, complicating the differentiation of MD from other severe psychiatric disorders.

Recent advances in genotyping and multimodal neuroimaging technologies have significantly improved data collection in large‐scale prospective cohort studies, facilitating the identification of genetic variants associated with brain imaging phenotypes using larger sample sizes than previously possible. Summary statistics from large‐scale genome‐wide association studies (GWAS) focusing on human brain morphology^[^ ^31^ ^]^ and functional networks^[^ ^32^ ^]^ allow for the systematic exploration of causal relationships between brain imaging phenotypes and the risk of MD through bidirectional MR analysis. Moreover, multivariable MR further enhances this approach by enabling the identification of core brain imaging phenotypes that may serve as predictive markers for MD onset.^[^ ^23^, ^33^, ^34^ ^]^ Here, we expand on previous research by employing bidirectional two‐sample MR analysis to assess causal relationships between 83 brain‐wide volumes, 191 resting‐state functional magnetic resonance imaging (rsfMRI) phenotypes, and MD or other severe psychiatric disorders with clinical commonality with MD including bipolar disorder, schizophrenia, schizotypal and delusional disorders, and ASD. We utilized multivariable MR to identify core brain imaging phenotypes associated with MD and the other psychiatric disorders. By comparing their differences, we elucidated the unique brain region volumes and functional networks associated with MD onset. In addition, to assess whether modifying common behaviors such as smoking, alcohol intake, and physical activity actively reduces the risk of developing MD, we applied potential predictive brain imaging phenotypes as mediators in a two‐step MR approach to quantify their mediating effects between these behaviors and the risk of MD onset. Our findings provide insights that may guide early‐stage prediction and prevention of MD through brain imaging.

## 2 Results

### 2.1 Overview of the Study Design

**Figure 1:**
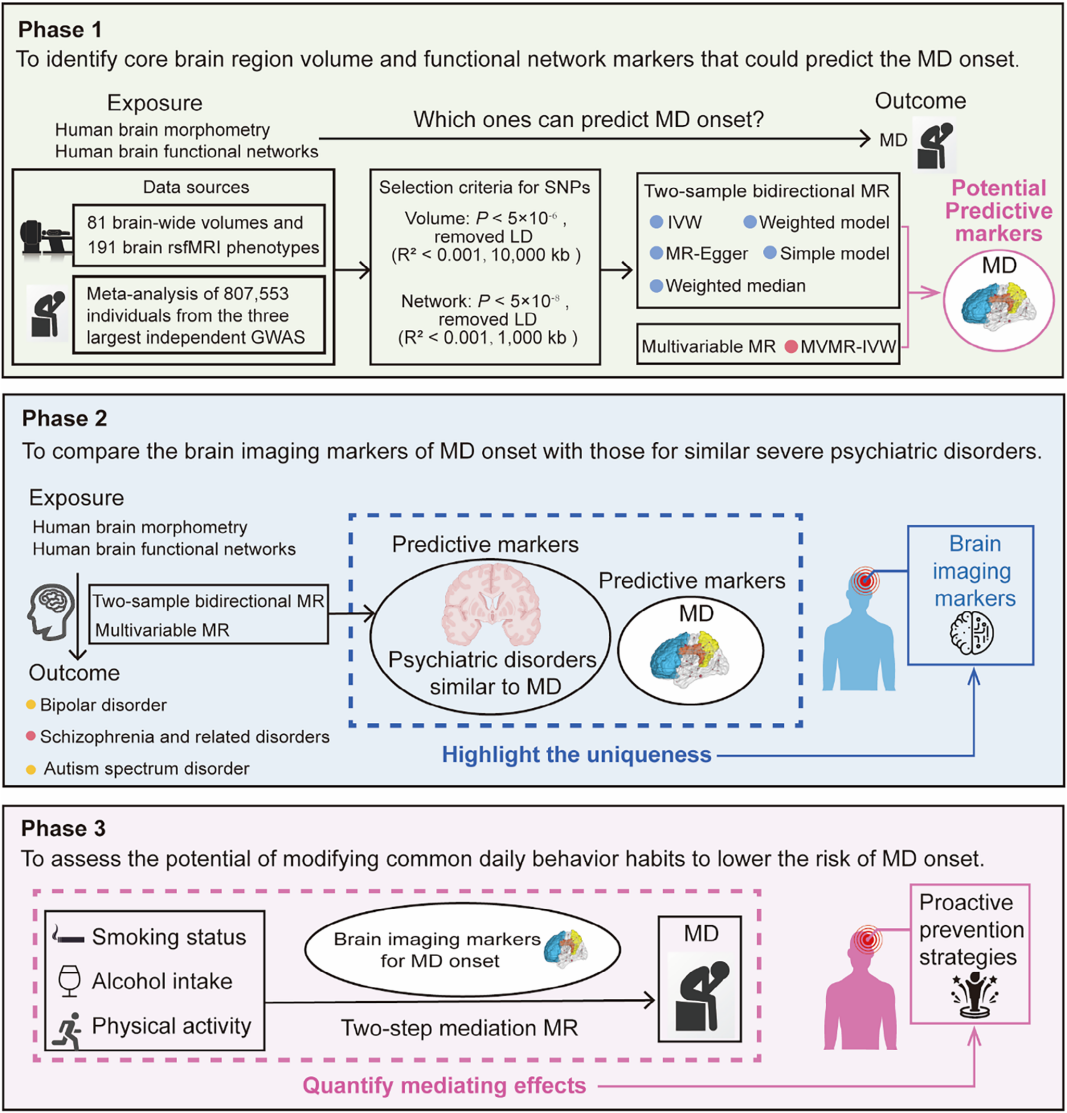
Overview of the MR study design. MD, major depression; rsfMRI, resting‐state functional magnetic resonance imaging; GWAS, genome‐wide association study; LD, linkage disequilibrium; SNPs, single nucleotide polymorphisms; MR, Mendelian randomization; IVW, inverse variance weighted; MVMR, multivariable MR.

As shown in **Figure** **1**, this study was conducted according to three‐phase analysis. In phase 1, we performed two‐sample bidirectional MR to explore potential causal relationships between 83 brain region volumes/191 brain functional networks and MD, identifying core brain imaging markers associated with its onset through multivariable MR. In phase 2, we compared core brain imaging markers between MD and other severe psychiatric disorders with shared clinical features, including bipolar disorder, schizophrenia, schizotypal disorder and delusional disorder, and ASD, to identify brain imaging markers potentially unique to MD onset. In phase 3, we used these unique potential markers as mediators in a two‐step MR approach to quantify their mediating effects on the causal relationship between common behaviors (smoking, drinking alcohol, various types of physical activity) and MD to assess the potential of modifying these behaviors to reduce the risk of MD onset.

### 2.2 Identification of Core Brain Region Volumes Associated with MD Onset

We first conducted bidirectional MR analyses to explore potential causal relationships between brain‐wide volumes and MD. The causal relationships between all 83 brain volumes and MD from the forward MR are detailed in File S2 (Supporting Information). The main inverse variance weighted (IVW) results from the forward MR indicated that the volumes of the left and right ventral diencephalon and thalamus were negatively associated with MD risk, and the direction of the MR‐Egger estimate was consistent with that of the IVW method (**Table** **1**). A one standard deviation (s.d.) decrease in left ventral diencephalon volume was associated with a 0.00014 s.d. increase in risk [odds ratio (OR) = 0.99986, 95% confidence interval (CI): 0.99979 to 0.99993, *P* = 0.00016, false discovery rate (FDR)‐adjusted *P* = 0.00451]. Similarly, a one s.d. decrease in right ventral diencephalon volume was associated with a 0.00018 s.d. increase in risk [OR = 0.99982, 95% CI: 0.99975 to 0.99989, *P* = 1.14393 × 10^−6^, FDR‐adjusted *P* = 0.00009], and a one s.d. decrease in thalamus volume was associated with a 0.00007 s.d. increase in risk [OR = 0.99993, 95% CI: 0.99989–0.99997, *P* = 0.00154, FDR‐adjusted *P* = 0.03196]. Linkage disequilibrium (LD) score regression analysis showed no genetic correlations between thalamus volume and MD. Cochran's Q statistic for the MR‐IVW analyses revealed no significant heterogeneity among IVs, and the MR Pleiotropy RESidual Sum and Outlier (MR‐PRESSO) global test and MR‐Egger intercept test detected no horizontal pleiotropy after excluding outliers, confirming the robustness of the forward MR results (Table S3, Supporting Information). Reverse MR analyses showed no evidence of a causal effect of MD on the identified brain region volumes (*P* > 0.05 for all), and sensitivity analyses indicated the robustness of the reverse MR results (Table S4, Supporting Information).

**Table 1:** Causalities in the forward MR analysis between identified brain region volumes and major depression.

Exposure	Outcome	Method	No. of SNP	Beta	SE	OR (95% CI)	*P*‐value	Adjusted *P*‐value
Left ventral diencephalon volume	Major depression	IVW	72	−0.00014	0.00004	0.99986 (0.99979–0.99993)	0.00016	0.00451
		MR‐Egger	72	−0.00015	0.00011	0.99985 (0.99964–1.00006)	0.17082	–
		Weighted median	72	−0.00011	0.00006	0.99989 (0.99978–1.00000)	0.04513	–
		Weighted mode	72	−0.00003	0.00012	0.99997 (0.99973–1.00021)	0.79783	–
		Simple mode	72	−0.00002	0.00015	0.99998 (0.99969–1.00026)	0.87406	–
Right ventral diencephalon volume		IVW	73	−0.00018	0.00004	0.99982 (0.99975–0.99989)	1.14393e‐06	0.00009
		MR‐Egger	73	−0.00022	0.00010	0.99978 (0.99958–0.99998)	0.03569	–
		Weighted median	73	−0.00014	0.00006	0.99986 (0.99975–0.99997)	0.01466	–
		Weighted mode	73	−0.00010	0.00012	0.99990 (0.99966–1.00014)	0.42067	–
		Simple mode	73	−0.00010	0.00015	0.99990 (0.99960–1.00021)	0.53571	–
Thalamus volume		IVW	71	−0.00007	0.00002	0.99993 (0.99989–0.99997)	0.00154	0.03196
		MR‐Egger	71	−0.00008	0.00007	0.99992 (0.99979–1.00005)	0.23314	–
		Weighted median	71	−0.00006	0.00003	0.99994 (0.99987–1.00000)	0.05134	–
		Weighted mode	71	−0.00011	0.00009	0.99989 (0.99971–1.00006)	0.20805	–
		Simple mode	71	−0.00008	0.00009	0.99992 (0.99974–1.00010)	0.38292	–

MR, mendelian randomization; IVW, inverse variance weighted; SNP, single nucleotide polymorphism; SE, standard error; OR, odds ratio; CI, confidence interval. “IVW”, “MR‐Egger”, “Weighted median”, “Weighted mode”, and “Simple mode” indicate MR analyses using these methods. To pursue a high level of precision in differential outcomes, this study meticulously retained the data to an accuracy of five decimal places. All statistical tests were two‐sided. The *P*‐values were adjusted for multiple testing; therefore, *P*‐value *<* adjusted *P*‐value < 0.05 was considered a significant association; *P*‐value < 0.05 < adjusted *P*‐value was considered a suggestive association.

Given the potential interrelationships between the identified brain region volumes, we conducted multivariable MR analysis, which accounts for interactions among brain region volumes, to estimate the direct contribution of individual brain region volume to the risk of MD onset. **Table** **2** shows that, compared with univariable models, the multivariable MR analysis resulted in the loss of statistical significance for previously observed causal genetic links between left ventral diencephalon volume [multivariable MR (MVMR)‐IVW OR = 0.99996, 95% CI: 0.99912–1.00079, *P* = 0.91913], right ventral diencephalon volume [MVMR‐IVW OR = 0.99990, 95% CI: 0.99901–1.00078, *P* = 0.82096], and thalamus volume [MVMR‐IVW OR = 0.99998, 95% CI: 0.99985–1.00012, *P* = 0.82779] and MD, after sequentially controlling for two other identified region volumes. We therefore further explored each of these three brain region volumes as the response variable. Variance inflation factor (VIF) analysis indicated VIF values <5 and condition number (κ) <10 (Table S5, Supporting Information) for each of the three analyses. These results support the conclusion that three identified brain regions have independent genetic influences without multicollinearity,^[^ ^35^, ^36^ ^]^ such that their distinct but synergistic alterations in volume may be associated with risk of MD onset.

**Table 2:** Multivariable MR results of effect estimate between core brain region volumes and major depression after adjusting for other identified brain region volumes.

Exposure	Adjustment of brain region volumes	Outcome	Method	Beta	SE	OR (95% CI)	*P*‐value
Left ventral diencephalon volume	Two other identified brain region volumes	Major depression	MVMR‐IVW	−0.00004	0.00043	0.99996 (0.99912–1.00079)	0.91913
Right ventral diencephalon volume			MVMR‐IVW	−0.00010	0.00045	0.99990 (0.99901–1.00078)	0.82096
Thalamus volume			MVMR‐IVW	−0.00002	0.00007	0.99998 (0.99985–1.00012)	0.82779

MVMR, multivariable Mendelian randomization; IVW, inverse variance weighted; SE, standard error; OR, odds ratio; CI, confidence interval. “MVMR‐IVW” indicate multivariable MVMR via the IVW method. To pursue a high level of precision in differential outcomes, this study meticulously retained the data to an accuracy of five decimal places. All statistical tests were two‐sided. A *P*‐value < 0.05 was considered significant.

### 2.3 Identification of Core Brain Functional Networks Associated with MD Onset

We next conducted bidirectional and multivariable MR analyses to identify core brain functional networks associated with MD onset. Causal relationship results between all 191 brain functional networks and MD from the forward MR are detailed in File S6 (Supporting Information). The main IVW results from the forward MR indicated that two rsfMRI phenotypes were negatively associated with MD risk (**Table** **3**). First, the activities of the precuneus gyrus, cuneus gyrus, or cingulate gyrus and frontal region were negatively associated with MD risk. The activity of these brain regions affected functional connectivity within the triple network (default mode network [DMN], central executive network [CEN], and salience network [SN]), with a one s.d. increase in the functional connectivity of these networks associated with a 16.906% reduction in MD risk [OR = 0.83094, 95% CI: 0.73435–0.94025, *P* = 0.00331, FDR‐adjusted *P* = 0.46380]. Second, the functional connectivity between the parietal and frontal regions was also negatively associated with MD risk. Specifically, a one s.d. increase in functional connectivity between the attention network and the triple network (CEN, SN, or DMN) was linked to an 11.572% decrease in MD risk [OR = 0.88428, 95% CI: 0.78700–0.99359, *P* = 0.00331, FDR‐adjusted *P* = 1.00000]. LD score regression analysis showed no genetic correlations between the functional connectivity of the parietal and frontal regions and MD. Sensitivity analyses revealed that with only two SNPs included, MR‐Egger intercept and MR‐PRESSO global tests could not be performed. Cochran's Q statistic from the MR‐IVW analyses showed no significant heterogeneity, indicating that our results were unaffected by heterogeneity bias (Table S7, Supporting Information). Findings of the reverse MR analyses did not detect any causal effect of MD on the identified rsfMRI phenotypes (*P* > 0.05 for all), and sensitivity analyses further confirmed the robustness of the reverse MR results (Table S8, Supporting Information).

**Table 3:** Causalities in the forward MR analysis between identified brain functional networks and major depression.

Exposure		Outcome	Method	No. of SNP	Beta	SE	OR (95% CI)	*P*‐value	Adjusted *P*‐value
Location	rsfMRI network								
edge_pheno1175 (Precuneus|Cuneus |Cingulate)&(Frontal)	(Default_mode| Central_executive)& (Salience|Default_mode)	Major depression	IVW	2	−0.18519	0.06305	0.83094 (0.73435–0.94025)	0.00331	0.46380
edge_pheno1269 (Parietal)&(Frontal)	(Central_executive| Attention)& (Central_executive| Salience|Default_mode)		IVW	2	−0.12298	0.05946	0.88428 (0.78700–0.99359)	0.00331	1.00000

MR, mendelian randomization; IVW, inverse variance weighted; SNP, single nucleotide polymorphism; SE, standard error; OR, odds ratio; CI, confidence interval; rsfMRI, resting‐state functional magnetic resonance images. “IVW” indicate MR analysis via the IVW method. To pursue a high level of precision in differential outcomes, this study meticulously retained the data to an accuracy of five decimal places. All statistical tests were two‐sided. The *P*‐values were adjusted for multiple testing; therefore, *P*‐value *<* adjusted *P*‐value < 0.05 was considered a significant association; *P*‐value < 0.05 < adjusted *P*‐value was considered a suggestive association.

We next used multivariable MR‐IVW to account for other identified rsfMRI phenotypes. **Table** **4** shows that the previously significant associations in functional connectivity between the parietal and frontal regions with MD became nonsignificant [MVMR‐IVW OR = 0.98062, 95% CI: 0.86210–1.11543, *P* = 0.76585] using multivariable MR‐IVW. However, significant associations were still detected for functional connectivity within the triple network (DMN or CEN and SN or DMN) affected by the activities of the precuneus gyrus or cuneus gyrus or cingulate gyrus and frontal region [MVMR‐IVW OR = 0.83362, 95% CI: 0.74557–0.93207, *P* = 0.00140]. This result suggests an independent causal effect of this network on MD, indicating that the functional connectivity within the triple network, influenced by the activities of these brain regions, may be associated with MD onset.

**Table 4:** Multivariable MR results of effect estimate between core brain functional networks and major depression after adjusting for other identified brain region volumes.

Exposure		Adjustment of brain functional networks	Outcome	Method	Beta	SE	OR (95% CI)	*P*‐value
Location	rsfMRI network							
edge_pheno1175 (Precuneus|Cuneus |Cingulate)&(Frontal)	(Default_mode| Central_executive)& (Salience|Default_mode)	The other identified brain functional network	Major depression	MVMR‐IVW	−0.18198	0.05695	0.83362 (0.74557–0.93207)	0.00140
edge_pheno1269 (Parietal)&(Frontal)	(Central_executive| Attention)& (Central_executive| Salience|Default_mode)			MVMR‐IVW	−0.01957	0.06572	0.98062 (0.86210–1.11543)	0.76585

MVMR, multivariable mendelian randomization; SE, standard error; OR, odds ratio; CI, confidence interval; IVW, inverse variance weighted; rsfMRI, resting‐state functional magnetic resonance images. “MVMR‐IVW” indicates MVMR via the IVW method. To pursue a high level of precision in differential outcomes, this study meticulously retained the data to an accuracy of five decimal places. All statistical tests were two‐sided. A *P*‐value < 0.05 was considered significant association.

### 2.4 Comparison of Core Brain Region Volumes between MD and Other Severe Psychiatric Disorders

**Figure 2:**
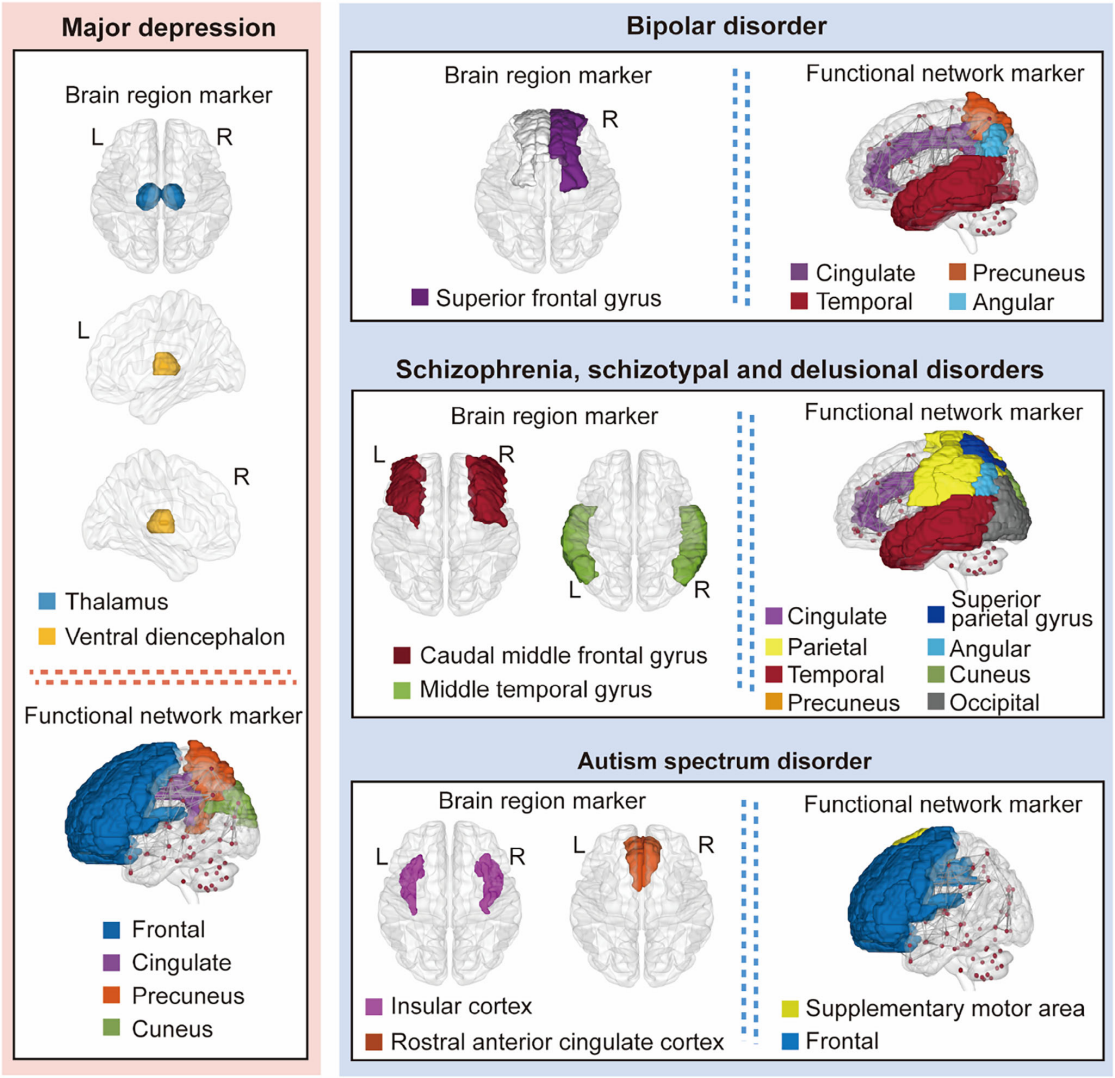
Comparison of core brain imaging markers associated with onset of major depression and other similar severe psychiatric disorders. Brain region volume and functional network markers (including the functional connectivity of involved brain regions) associated with the onset of major depression and other similar severe psychiatric disorders. Markers were identified through two‐sample bidirectional MR and multivariable MR screening. MR, Mendelian randomization; L, left cerebral hemisphere; R, right cerebral hemisphere.

We next applied bidirectional and multivariable MR to discriminate core brain region volumes in common severe psychiatric disorders with clinical overlap with MD, and in doing so detected distinct brain region volumes specific to these psychiatric disorders and different from those associated with MD (**Figure** **2**). The causal relationship results between all 83 brain volumes and other severe psychiatric disorders (bipolar disorder, schizophrenia, schizotypal and delusional disorders, and ASD) using forward MR are detailed in File S2 (Supporting Information). Compared to MD, the main IVW analysis in the forward MR indicated 1) significant causal relationships between volume changes in the left and right superior frontal gyri, entorhinal cortex, and posterior cingulate cortex and bipolar disorder; 2) variations in volume within the caudal anterior cingulate cortex, caudal middle frontal gyrus, fusiform gyrus, and middle temporal gyrus, suggesting potential causal connections with schizophrenia, schizotypal disorder, and delusional disorder; 3) volume changes in the insular cortex, nucleus accumbens, and rostral anterior cingulate cortex, suggesting potential causal associations with ASD (File S9, Supporting Information). LD score regression analysis showed no significant genetic correlations between the identified brain region volumes and these severe psychiatric disorders, similar to MD. In addition, sensitivity analyses of the causal associations from the main IVW analysis further confirmed the robustness of these results (Table S3, Supporting Information). In reverse MR analysis, using these severe psychiatric disorders as exposures showed no causal effects on the associated brain region volumes, with further sensitivity analyses confirming the reliability of the reverse MR results (File S10, Supporting Information).

Subsequently, multivariable MR analysis showed that, after adjusting for other identified brain region volumes, significant causal associations remained for the right superior frontal gyrus volume [MVMR‐IVW OR = 1.00015, 95% CI: 1.00001–1.00028, *P* = 0.03359] with bipolar disorder; middle temporal gyrus volume [MVMR‐IVW OR = 1.00014, 95% CI: 1.00001–1.00028, *P* = 0.03932] with schizophrenia, schizotypal, and delusional disorders; and insular cortex volume [MVMR‐IVW OR = 1.00024, 95% CI: 1.00005–1.00044, *P* = 0.01367] and rostral anterior cingulate cortex volume [MVMR‐IVW OR = 0.99945, 95% CI: 0.99893–0.99996, *P* = 0.03598] with ASD (Table S11, Supporting Information). These regions are proposed as core markers for these severe psychiatric disorders.

### 2.5 Comparison of Core Brain Functional Networks between MD and Other Common Severe Psychiatric Disorders

We next conducted bidirectional and multivariable MR to identify core brain functional networks that discriminate common severe psychiatric disorders from MD. The predictive rsfMRI phenotypes for MD onset were different to those associated with other severe psychiatric disorders (Figure 2). The causal relationship results between all 191 brain functional networks and the other severe psychiatric disorders from the forward MR are detailed in File S6 (Supporting Information). Compared to MD, the main IVW analysis in the forward MR indicated a significant causal link between altered functional connectivity in the triple network (CEN, SN, or DMN) and bipolar disorder, where the triple networks are linked to activity in the temporal regions (notably the middle temporal region), frontal regions (notably the inferior frontal, superior frontal, and supplementary motor area), as well as the precuneus, angular, and cingulate regions. For schizophrenia, schizotypal, and delusional disorders, causal associations were suggested by altered functional connectivity in the triple network (CEN, SN, or DMN) and attention and visual networks, which affect connectivity between the precuneus, angular, cingulate, cuneus, temporal, occipital, and superior parietal regions. In ASD, altered connectivity in the triple network (CEN, SN, or DMN), motor, subcortical‐cerebellum, and limbic networks suggested causal links. These networks are related to frontal region activity (predominantly inferior frontal, superior frontal, and supplementary motor area), as well as the postcentral, precentral, paracentral, insula, cingulate, and cerebellum regions (File S12, Supporting Information). LD score regression analysis revealed potential genetic correlations only for functional connectivity within the triple network (CEN, SN, or DMN), influenced by the supplementary motor area in the frontal region with bipolar disorder and the subcortical‐cerebellar network with ASD. Sensitivity analyses of the IVW results showed no significant heterogeneity nor horizontal pleiotropy, confirming the robustness of these findings (Table S7, Supporting Information). Reverse MR analysis using these severe psychiatric disorders as exposures found no causal effects on the identified rsfMRI phenotypes, with sensitivity analyses supporting these reverse MR results (File S13, Supporting Information).

Extended multivariable MR analysis identified core brain functional networks related to severe psychiatric disorders and revealed that, even after adjusting for other identified rsfMRI phenotypes, there were significant causal associations between the functional connectivity of DMN and CEN [MVMR‐IVW OR = 1.31567, 95% CI: 1.07313–1.61303, *P* = 0.00832] related to the activity of precuneus or angular or cingulate and temporal regions and bipolar disorder. Interestingly, for schizophrenia, schizotypal, and delusional disorders, all previously identified rsfMRI phenotypes lost their statistical significance [MVMR‐IVW *P* > 0.05 for all]. With respect to ASD, there was a significant association between the functional connectivity of the SN and DMN [MVMR‐IVW OR = 0.66585, 95% CI: 0.48018–0.92329, *P* = 0.01475], influenced by the activity of the supplementary motor area in the frontal region (Table S14, Supporting Information). These findings suggest that the onset of bipolar disorder and ASD is associated with specific rsfMRI phenotypes that serve as core markers. In contrast, the rsfMRI phenotypes associated with schizophrenia, schizotypal disorder, and delusional disorders appear to exert an synergistic influence rather than isolated impacts from individual rsfMRI phenotypes.

### 2.6 Insights from Assessing Alterations in Core Brain Imaging Phenotypes to Explore Potential Preventative Modifications in Common Behaviors to Prevent MD Onset

First, we conducted forward MR to investigate the potential causal effects of common behaviors on MD risk. The main IVW analysis indicated that, among the autonomously engaged daily behaviors of smoking status, alcohol frequency weekly, alcohol drinker status, and various types of physical activity over a 4‐week period, decreased smoking status [OR = 1.71100, 95% CI: 1.45762–2.00843, *P* = 5.09903 × 10^−11^], increased alcohol frequency weekly [OR = 0.98219, 95% CI: 0.96861–0.99596, *P* = 0.01139], alcohol drinker status [OR = 0.81649, 95% CI: 0.68182–0.97776, *P* = 0.02749], and engagement in physical activities such as walking for leisure [OR = 0.81760, 95% CI: 0.73679–0.90727, *P* = 0.00015] and other exercises (e.g., swimming, cycling, keep fit, bowling) [OR = 0.74415, 95% CI: 0.66599–0.83147, *P* = 1.78881 × 10^−7^] had potential causal preventive effects on MD risk. Sensitivity analyses confirmed the robustness of these findings (File S15, Supporting Information).

Second, we conducted forward MR to investigate the potential causal effects of common behaviors on the core brain region volumes and functional networks associated with MD onset. The main IVW analysis showed that each one s.d. increase in smoking status was significantly associated with decreased volumes in the left ventral diencephalon [OR = 3.61098 × 10^−62^, 95% CI: 1.08302 × 10^−96^ to 1.20396 × 10^−27^, *P* = 0.00049] and right ventral diencephalon [OR = 5.17865 × 10^−59^, 95% CI: 1.37778 × 10^−92^ to 1.94649 × 10^−25^, *P* = 0.00067], as well as reduced functional connectivity within the triple network (DMN or CEN and SN or DMN) affected by the activities of the precuneus gyrus, cuneus gyrus, or cingulate gyrus and frontal region [OR = 0.71800, 95% CI: 0.56103–0.91888, *P* = 0.00848]. Similarly, each one s.d. increase in genetically determined alcohol frequency weekly was associated with increased functional connectivity of this core rsfMRI phenotype [OR = 1.02166, 95% CI: 1.00259–1.04109, *P* = 0.02579], while each one s.d. increase in genetically determined alcohol drinker status was associated with decreased thalamus volume [OR = 5.24623 × 10^−53^, 95% CI: 5.61940 × 10^−103^ to 0.00490, *P* = 0.04031]. Moreover, a one s.d. increase in walking for leisure over a four‐week period was associated with increased thalamus volume [OR = 3.29652 × 10^−37^, 95% CI: 82828.27814 to 1.31199 × 10^70^, *P* = 0.02409]. Similarly, a one s.d. increase in engagement in other exercises (e.g., swimming, cycling, keep fit, bowling) over a four‐week period was associated with increased thalamus volume [OR = 4.96917 × 10^75^, 95% CI: 5.26345 × 10^34^ to 4.69134 × 10^116^, *P* = 0.00029] and right ventral diencephalon volume [OR = 7.92413 × 10^29^, 95% CI: 62642.85289 to 1.00238 × 10^55^, *P* = 0.01957]. Again, sensitivity analyses confirmed these findings (File S15, Supporting Information).

**Figure 3:**
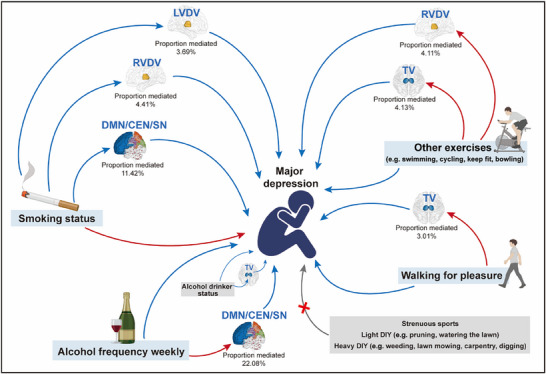
Mediation analysis of the effects of daily behavioral habits on major depression via unique potential predictive brain imaging phenotypes. Blue arrows indicate the initial variable that significantly decreases the risk associated with the tail variable. Red arrows represent the initial variables that significantly increase the risk associated with the tail variable. The gray arrow indicates the initial variable that does not significantly alter the risk associated with the tail variable. The blue box highlights that specific regular behaviors can influence the risk of major depression onset through unique potential predictive brain region volume and/or functional networks, while the gray box suggests that specific behaviors do not affect the risk of major depression onset or influence it through unique potential predictive brain imaging phenotypes. LVDV, left ventral diencephalon volume; RVDV, right ventral diencephalon volume; TV, thalamus volume; DMN, default mode network; CEN, central executive network; SN, salience network; DIY, do it yourself.

Finally, considering behaviors such as smoking status, alcohol frequency weekly, alcohol drinker status, walking for leisure, and other exercises over a four‐week period, these habits showed a genetic predisposition linked to the risk of MD and its associated predictive core brain region volume and functional network markers. We conducted two‐step MR to explore whether changes in these habits influenced the risk of MD onset through alterations in these core markers. **Figure** **3** and **Table** **5** show that smoking status indirectly affected MD by modulating core brain region volumes and functional networks, with the following mediation effects: left ventral diencephalon volume 0.01981 (95% CI: 0.00656–0.03721), with a mediation proportion of 3.68862%; right ventral diencephalon volume 0.02369 (95% CI: 0.00876–0.04237), with a mediation proportion of 4.41124%; and functional connectivity within the triple network (DMN or CEN and SN or DMN) influenced by the activities of the precuneus gyrus or cuneus gyrus or cingulate gyrus and frontal region 0.06135 (95% CI: 0.00968–0.13452), accounting for a mediation proportion of 11.42327%. Alcohol frequency weekly also indirectly affected MD as functional connectivity within the triple network (DMN or CEN and SN or DMN) was influenced by the activities of the precuneus gyrus, cuneus gyrus, or cingulate gyrus and frontal region −0.00397 (95% CI: −0.00924 to −0.00030), accounting for a mediation proportion of 22.07802%.

**Table 5:** Mediation effect of daily behaviors on major depression through effects on core brain region volume and potential functional network markers associated with risk of major depression onset.

Exposure	Mediator	Total effect C *β* (95% CI)	Direct effect A *β1* (95% CI)	Direct effect B *β2* (95% CI)	Mediation effect *β3* (95% CI)	Mediated proportion (%)
Smoking status	Left ventral diencephalon volume	1.71100 (1.45762–2.00843)	−141.47630 (−220.96841 to −61.98418)	−0.00014 (−0.00021 to −0.00007)	0.01981 (0.00656–0.03721)	3.68862%
	Right ventral diencephalon volume		−134.20798 (−211.51735 to −56.89860)	−0.00018 (−0.00025 to −0.00011)	0.02369 (0.00876–0.04237)	4.41124%
	(Default_mode| Central_executive)& (Salience| Default_mode)		−0.33129 (−0.57797 to −0.08460)	−0.18519 (−0.30878 to −0.06161)	0.06135 (0.00968–0.13452)	11.42327%
Alcohol frequency weekly	(Default_mode| Central_executive)& (Salience| Default_mode)	−0.01797 (−0.03190 to −0.00405)	0.02142 (0.00259–0.04027)	−0.18519 (−0.30878 to −0.06161)	−0.00397 (−0.00924 to −0.00030)	22.07802%
Alcohol drinker status	Thalamus volume	−0.20274 (−0.38299 to −0.02249)	−120.37950 (−235.44004 to −5.31896)	−0.00007 (−0.00011 to −0.00003)	–	–
Physical activities over 4 weeks: walking for pleasure	Thalamus volume	−0.20138 (−0.30545 to −0.09732)	86.38851 (11.32452–161.45250)	−0.00007 (−0.00011 to −0.00003)	−0.00605 (−0.01374 to −0.00057)	3.00624%
Physical activities over 4 weeks: other exercises (e.g., swimming, cycling, keep fit, bowling)	Thalamus volume	−0.29552 (−0.40648 to −0.18456)	174.29713 (79.94868–268.64559)	−0.00007 (−0.00011 to −0.00003)	−0.01222 (−0.02383 to −0.00351)	4.13332%
	Right ventral diencephalon volume		68.84488 (11.04520–126.64456)	−0.00018 (−0.00025 to −0.00011)	−0.01215 (−0.02482 to −0.00182)	4.11253%

CI, confidence interval. “Total effect C β” refers to the effect of daily behavioral habit on major depression; “Direct effect A β1” refers to the effect of daily behavioral habit on core brain region volume or functional networks; “Direct effect B β2” refers to the effect of core brain region volume or functional networks on major depression; “Mediation effect β3” refers to the effect of daily behavioral habit on major depression through core brain region volume or brain functional networks. Total effect and direct effects A and B were derived using the inverse variance weighted method, while the mediation effect was derived using the delta method. To pursue a high level of precision in differential outcomes, this study meticulously retained the data to an accuracy of five decimal places. All statistical tests were two‐sided. A *P*‐value < 0.05 was considered significant.

Walking for leisure over a four‐week period influenced MD by modulating thalamus volume, with a mediation effect estimated at −0.00605 (95% CI: −0.01374 to −0.00057), accounting for 3.00624% of the mediation proportion. Other exercises over a four‐week period indirectly affected MD by modulating core brain volumes, with the following mediation effects: thalamus volume showed a mediation effect of −0.01222 (95% CI: −0.02383 to −0.00351), accounting for 4.13332% of the mediation proportion; and the right ventral diencephalon volume had a mediation effect of −0.01215 (95% CI: −0.02482 to −0.00182), contributing 4.11253% to the mediation proportion.

Alcohol drinker status was negatively correlated with thalamus volume, so quantifying the mediating effects between alcohol drinker status and MD using a two‐step MR approach was not possible. These findings suggest that modifications to daily habits, including quitting smoking, increasing alcohol frequency moderately each week (without developing alcohol use disorder), engaging in leisure walking, and increasing participation in other exercises like swimming, cycling, keep fit, and bowling can progressively influence core brain regions and functional networks, potentially reducing the risk of MD onset.

## 3 Discussion

Here, by using genetic variants as reliable proxies for human brain morphometry^[^ ^31^ ^]^ and functional networks,^[^ ^32^ ^]^ we performed bidirectional two‐sample MR analyses to systematically investigate causal relationships between 83 brain region volumes, 191 fMRI phenotypes, and MD and then employed multivariable MR to identify core brain region volumes and functional networks associated with MD onset. Our results show that, unlike other similar severe disorders such as bipolar disorder, schizophrenia, schizotypal and delusional disorders, and ASD, reductions in the volumes of the left and right ventral diencephalon and thalamus, along with decreased functional connectivity between the precuneus gyrus, cuneus gyrus, or cingulate gyrus and frontal regions within the triple network (DMN, SN and CEN), serve as unique brain imaging markers for MD onset.

Previous MR studies have explored causal relationships between changes in brain region volumes or structures and MD.^[^ ^25^, ^26^, ^27^, ^28^, ^29^, ^30^ ^]^ However, these studies usually relied on univariable MR to focus on broader associations without thoroughly examining the specific changes in core brain region volumes that significantly influence MD onset. This knowledge gap underscores the need for more targeted investigations that clarify the precise relationships between specific brain volume changes and disease risk. In our study, we utilized bidirectional and multivariable MR to identify brain region volumes that potentially predict MD onset. First, our forward MR analysis revealed that left ventral diencephalon, right ventral diencephalon, and thalamus volumes were negatively associated with MD. Importantly, reverse MR analysis showed no evidence of a causal effect of MD on the volumes of these brain regions, indicating that these volumes are solely related to MD onset. When multivariable MR analysis was used to estimate the direct contributions of each brain region while accounting for the effects of other identified regions, we found that the changes in the volumes of these specific regions could not be isolated with respect to their impact on MD onset. Additionally, VIF analysis confirmed independent genetic influences of these brain volumes with no multicollinearity, supporting the hypothesis that volumetric reductions in these three core regions may synergistically contribute to MD onset.

The ventral diencephalon, which includes the hypothalamus and thalamus, plays a crucial role in regulating important physiological processes such as hormone secretion, sensory processing, and emotional responses, thereby significantly influencing behavior and mental health.^[^ ^37^ ^]^ Specifically, atrophy of hypothalamic nuclei may impair glucocorticoid receptor‐mediated negative feedback mechanisms, resulting in sustained hypothalamic‐pituitary‐adrenal (HPA) axis hyperactivity—a well‐characterized pathophysiological feature of depression.^[^ ^38^, ^39^, ^40^, ^41^ ^]^ This glucocorticoid excess induces hippocampal neurotoxicity, impairs synaptic plasticity, and dysregulates monoaminergic neurotransmission, particularly within serotonin (5‐HT) and dopaminergic pathways, thereby contributing to maladaptive emotional processing and symptom manifestation.^[^ ^41^, ^42^ ^]^ In addition, recent advances in circuit neuroscience have demonstrated that the ventral diencephalon is involved in stress‐responsive neural networks through distinct projection pathways. Chronic stress exposure induces hyperactivity in lateral hypothalamus (LH) neurons projecting to the lateral habenula (LHb), driving synaptic potentiation that encodes aversive experiences and promotes depression‐like behaviors.^[^ ^43^ ^]^ Notably, social defeat paradigms demonstrate that LH neurons generate negative prediction errors during unexpected social loss, with LH‐LHb circuit activation suppressing medial prefrontal cortex (mPFC) activity to reinforce social withdrawal and depressive phenotypes.^[^ ^44^ ^]^ Another study identified the anterior paraventricular thalamus (aPVT) as a nodal structure mediating heterogeneous depression‐like responses to chronic stress. The mPFC‐aPVT pathway appears to coordinate behavioral subtype specification, particularly the co‐occurrence of social withdrawal and anhedonia.^[^ ^45^ ^]^ These structural‐functional alterations may establish a self‐reinforcing pathogenic cycle: diencephalic degeneration increases neural vulnerability to stress exposure, which subsequently accelerates structural deterioration through glucocorticoid‐mediated neurotoxicity and circuit‐level maladaptation. This reciprocal relationship between morphological changes and functional dysregulation potentially underlies both disease progression and treatment resistance in MD.

Simultaneously, as relationships between abnormalities in brain structure and function are nonlinear, structural alterations may not equate to a change in function. Current evidence suggests that impairments in brain functional networks often occur before or at the same time as structural modifications in specific brain regions during the emergence of depression.^[^ ^46^, ^47^, ^48^ ^]^ This also suggests that early detection of functional network disturbances, combined with alterations in the specific brain region volumes, may be crucial for predicting MD onset. To our knowledge, only one MR study has explored the causal relationship between brain functional networks and depression, reporting that abnormalities in the functional connectivity of the DMN and CEN were associated with the risk of developing depression.^[^ ^49^ ^]^ However, this study only used forward MR analysis and did not employ reverse or multivariable approaches to identify unique brain functional network markers. In addition, they did not further investigate the relationship between these functional networks and individuals with MD. We found that decreased functional connectivity between the precuneus gyrus, cuneus gyrus, or cingulate gyrus and frontal regions within the triple network (DMN, SN and CEN) may be associated with the onset of MD based on bidirectional and multivariable MR analyses.

The precuneus gyrus is located in the medial parietal lobe, and dysfunction of this area is associated with negative self‐referential thinking and rumination.^[^ ^50^, ^51^ ^]^ Dysfunction of the cuneus gyrus, situated in the posterior occipital lobe, can disrupt visual attention, leading to an increased focus on negative stimuli and exacerbating depressive symptoms.^[^ ^52^, ^53^ ^]^ Dysfunction of the cingulate gyrus, located above the corpus callosum, can increase sensitivity to emotional pain and impair emotional responses, contributing to feelings of hopelessness and difficulty concentrating in individuals with depression.^[^ ^54^, ^55^ ^]^ Altered frontal activity is commonly observed in individuals with depression, leading to difficulties in decision‐making and emotional management.^[^ ^56^, ^57^ ^]^ Numerous clinical investigations have also validated our findings, reporting that reduced activity in these brain regions or decreased functional connectivity between the precuneus, cingulate gyrus, and frontal regions is closely linked to a higher risk of developing depression.^[^ ^50^, ^52^, ^54^, ^55^, ^56^, ^57^, ^58^ ^]^

The DMN, CEN, and SN are three critical brain networks that play distinct yet interconnected roles in cognitive and emotional processing.^[^ ^32^ ^]^ Interactions between the DMN, CEN, and SN are essential for effective emotional and cognitive functioning.^[^ ^32^ ^]^ Disrupted connectivity between the DMN and CEN can hinder the ability to shift attention away from negative self‐referential thoughts, worsening depressive symptoms.^[^ ^59^, ^60^ ^]^ Similarly, reduced connectivity between the SN and CEN can impair focus on salient information, resulting in cognitive biases and emotional dysregulation, while diminished connectivity between the DMN and SN may lead to excessive preoccupation with negative thoughts and memories, undermining the adaptive regulation typically provided by the SN and reinforcing depressive states.^[^ ^59^, ^60^ ^]^

After identifying core brain region volume and functional network markers associated with MD onset through bidirectional and multivariable MR analyses, we then used the same methodology to evaluate predictive factors for other prevalent, similar severe psychiatric disorders including bipolar disorder, schizophrenia, schizotypal and delusional disorders, and ASD. We compared the brain region volumes and functional networks potentially predicting MD with those associated with these psychiatric disorders to determine whether the identified markers could reliably be associated with the risk of developing MD. With respect to brain region volume, the right superior frontal gyrus was a primary marker for the onset of bipolar disorder, while the caudal middle frontal gyrus and middle temporal gyrus were key markers for schizophrenia, schizotypal, and delusional disorders. In addition, the insular cortex and rostral anterior cingulate cortex were significant markers for ASD. These findings suggest that the brain region volumes potentially predicting the onset of each of these disorders are unique, consistent with the current clinical evidence,^[^ ^61^, ^62^, ^63^, ^64^ ^]^ and highlight their clinical significance. Our findings on functional network connectivity reveal that these psychiatric disorders share predictive patterns with those associated with MD, with all associated with abnormalities in the functional connectivity of the DMN, CEN, and SN. However, there were also notable differences in the specific brain regions involved in affecting these functional networks. For example, reduced functional connectivity between the precuneus gyrus, angular gyrus, or cingulate gyrus and the temporal region was associated with a lower risk of developing bipolar disorder, while changes in functional connectivity across multiple brain regions, including the parietal, temporal, and occipital lobes, were associated with the risk of schizophrenia, schizotypal disorder, and delusional disorders. Furthermore, decreased activity in the supplementary motor area of the frontal regions was associated with a higher risk of ASD.

This comparative analysis further suggests that two key factors may serve as distinct predictive indicators for MD onset: first, reductions in the volumes of the left and right ventral diencephalon and thalamus; and second, decreased functional connectivity between the precuneus gyrus, cuneus gyrus, or cingulate gyrus and frontal regions within the triple network (DMN, SN, and CEN). A growing body of clinical brain imaging evidence from diverse ethnic populations, not only of European ancestry but also Asian and American populations, demonstrates that untreated first‐episode MD is associated with reduced raw thalamus and ventral diencephalon volumes.^[^ ^37^ ^]^ This structural pattern appears particularly pronounced in early‐onset MD, where volumetric deficits in both regions correlate with disease severity and progression.^[^ ^65^, ^66^ ^]^ Furthermore, exposure to childhood trauma and adversity exacerbates volume loss in these regions,^[^ ^67^ ^]^ with independent effects on suicidal ideation and cognitive dysfunction.^[^ ^65^ ^]^ These structural alterations are increasingly recognized as biomarkers of lifetime MD susceptibility, potentially reflecting cumulative neuroplastic adaptations to chronic stress.^[^ ^68^ ^]^ In addition, significant differences in functional connectivity patterns within the triple network (DMN, SN, and CEN) have been observed between MD patients and healthy controls across ethnic groups.^[^ ^69^, ^70^ ^]^ Improvements in resting‐state functional connectivity, particularly in networks involved in reward processing and emotional regulation, correlate with clinical depressive symptom remission.^[^ ^71^, ^72^, ^73^ ^]^ Critically, effective prevention not only mitigates volume loss but also normalizes aberrant functional connectivity, and this dual restorative effect—structural volume recovery and network connectivity stabilization—potentially interrupts the exacerbation of depressive disorder symptoms and provides rapid antidepressant effects.^[^ ^71^, ^72^, ^73^, ^74^, ^75^ ^]^ Therefore, alterations in combined structural (thalamus and ventral diencephalon volumes) and functional (triple network connectivity) imaging phenotypes may serve as unique markers for the potential prediction of MD onset.

The serious and treatment‐resistant nature of MD mandates early prevention strategies before the condition fully develops. While numerous studies have shown that daily behaviors such as smoking cessation,^[^ ^76^ ^]^ alcohol consumption,^[^ ^77^ ^]^ and increased physical activity^[^ ^78^ ^]^ can help to reduce depressive symptoms, it remains unclear whether these changes effectively reduce the risk of MD onset. Therefore, we used the identified potential predictive brain imaging phenotypes as mediators to further explore causal relationships between modifications in behaviors and the risk of MD onset. First, our results indicate that stopping smoking could reduce the risk of MD onset through the identified brain imaging markers, especially via connectivity of core brain functional networks within the triple network (DMN, SN and CEN). Previous studies have shown that smoking exacerbates neuroinflammation and oxidative stress, adversely affecting brain structure and function.^[^ ^79^ ^]^ Nicotine, a key component of tobacco, interacts with neurotransmitter systems, particularly serotonin and dopamine, disrupting their balance and altering brain connectivity and volume.^[^ ^79^ ^]^ Second, we observed that alcohol intake may reduce the risk of developing MD. Increased weekly alcohol frequency could promote the connectivity of core functional networks within the triple network (DMN, SN, and CEN); however, long‐term alcohol misuse may potentially reduce thalamus volume. Alcohol‐related mood improvements likely originate from limbic system modulation through dopamine activation and glutamatergic inhibition, while prolonged ethanol consumption induces HPA axis hyperactivity, brain degeneration, and monoamine depletion, which are pathophysiological mechanisms characteristic of mood disorders.^[^ ^77^, ^80^, ^81^ ^]^ Third, we found that: 1) increased leisurely walking over a 4‐week period was associated with increased thalamic volume; 2) greater participation in exercises such as swimming, cycling, keep fit, and bowling correlated with increased thalamus and right ventral diencephalon volumes, potentially reducing the risk of MD onset. These findings are consistent with previous studies indicating that regular physical activity improves mood and reduces stress by promoting neurogenesis and enhancing brain plasticity.^[^ ^82^, ^83^ ^]^ However, when unique brain imaging markers were used as mediators to assess the impact of daily behaviors (such as smoking, alcohol intake, and physical activity) on the risk of MD onset, we observed that the mediation effects for prevention of MD attributable to changes in core brain region volumes were only 3%–5%, indicating that numerous unknown biological pathways influence the development of MD. Future studies should integrate multi‐omics approaches, including epigenetic and proteomic data, to identify additional mediating factors, which could facilitate the development of novel intervention strategies for MD prevention and treatment. Despite the limited magnitude of these mediation effects, our findings highlight the importance of incorporating behavioral modifications into public health strategies to proactively prevent MD through population‐level interventions.

This MR study is strengthened by the inclusion of a comprehensive and representative collection of available GWASs. The human brain morphometry reflects the latest advances in the field, and the analysis of brain functional networks includes the largest set of rsfMRI phenotypes to date. Second, the data on MD were derived from a meta‐analysis of the three largest independent GWAS on depression. Third, additional data sources were obtained from various representative databases, such as the FinnGen Consortium and the Psychiatric Genomics Consortium (PGC), further strengthening our findings. We strictly adhered to the Strengthening the Reporting of Observational Studies in Epidemiology Using MR (STROBE‐MR) checklist,^[^ ^84^, ^85^ ^]^ and we employed bidirectional and multivariable MR to establish normative criteria for screening, identifying, and differentiating MD from other similar severe psychiatric disorders. This approach ultimately enabled us to identify unique brain imaging markers of MD onset by examining the volumes of specific brain regions and the degree of intrinsic and stable functional network connectivity of the human brain. The robustness of these results was confirmed through Benjamini–Hochberg FDR correction for multiple testing, genetic correlation analysis, VIF analysis, and assessments of heterogeneity and horizontal pleiotropy. Finally, we utilized these unique brain imaging markers as mediators in a two‐step MR analysis to verify that common behavioral modifications such as smoking cessation, moderate alcohol intake, and increased physical activity might reduce the risk of MD onset. This perspective highlights the significance of proactive strategies for the early prevention of MD.

Our findings must, however, be interpreted with caution with respect to clinical application, as our study has limitations. First, despite selecting strongly associated SNPs, the genetic variants explained only a small fraction of the total variance and cannot be considered exact proxies of exposure.^[^ ^23^, ^24^ ^]^ Second, since we do not yet fully understand the biological mechanisms related to the genetic instruments, we cannot completely rule out potential violations of the independence and exclusion restriction assumptions, particularly regarding pleiotropy.^[^ ^23^, ^24^ ^]^ Third, nearly all existing MR methods rely on several fundamental assumptions, and genetic variation may not fully capture the complexities of environmental changes.^[^ ^33^, ^34^ ^]^ Moreover, MR estimates reflect lifelong genetic predispositions rather than the effects of modifiable interventions.^[^ ^33^, ^34^ ^]^ These limitations and the approach, which is primarily based on statistical inference, could introduce bias. Fourth, although similar clinical evidence has been found in populations outside of those of European descent, such as Asian and American populations, our study is limited by the fact that all summary‐level GWAS data were obtained from individuals of European ancestry from consortia or cohort studies. Therefore, caution should be exercised when extrapolating our findings to other ethnic populations. Fifth, our estimates of causal effects might be affected by winner's curse bias, which arises when the same dataset is used to choose both genetic variants as instrumental variables and to assess their relationship with the exposure.^[^ ^86^ ^]^ Although using separate, non‐overlapping datasets can help to reduce this bias, it also significantly decreases sample sizes, creating a trade‐off between the risk of bias and the precision of our estimates. Nevertheless, it is comforting to note that in large samples, like those from the UK Biobank, the influence of this bias is generally minimal and is unlikely to change the direction or strength of our findings.^[^ ^86^ ^]^ To address these limitations, additional, high‐quality clinical longitudinal assessments are essential for evaluating the dynamic changes in brain imaging phenotypes linked to the onset and progression of MD. It is also important to consider broader preventive factors associated with dietary and lifestyle variables. Furthermore, future research should establish genetic databases that include more diverse populations, such as those from America, Asia, and Africa, and conduct analyses that account for sex differences. This comprehensive approach will enhance our understanding of how these markers can predict serious mental illness outcomes and guide the development of more effective prevention strategies.

In conclusion, we conducted the first bidirectional and multivariable MR analyses using large‐scale GWAS data to identify core brain region volume and functional network markers associated with the risk of MD onset. By employing these unique brain imaging markers as mediators in a two‐step mediation MR analysis, we demonstrate how modified behaviors can contribute to the prevention of MD. These findings enhance our understanding of potential predictive outcomes at the brain imaging level and support proactive prevention strategies through behavioral modifications aimed at reducing the risk of severe mental health disorders.

## 4 Experimental Section

### Ethical Approval

The study followed the STROBE‐MR checklist,^[^ ^84^, ^85^ ^]^ as detailed in Table S16 (Supporting Information). All summary‐level GWAS data used in the analyses are publicly available. Since ethical approval was obtained for each of the original GWAS studies, this study did not require separate ethical review or approval.

### Data Sources

To minimize the possibility of confounding due to population stratification, all summary‐level GWAS data used were derived from individuals of European descent from reliable consortia or studies. Further information about GWAS data sources is summarized in Table S17 (Supporting Information).

### Human Brain Morphometry

Brain morphometry datasets were sourced from research by Fürtjes et al.,^[^ ^31^ ^]^ representing the latest GWAS on human brain morphometry. This study utilized genomic principal component analysis to model brain‐wide morphometry based on genetic architecture, applying it to genome‐wide association data for 83 cortical and subcortical gray matter volumes from European individuals in the UK Biobank (*n* = 36 778). The volumes included 33 cortical Desikan‐Killiany regions and 8 subcortical regions per hemisphere, along with the brain stem. Detailed information on the brain morphometry phenotypes is available in the original publication and Table S18 (Supporting Information).

### Human Brain Functional Networks

The rsfMRI datasets used here, acquired from the study by Zhao et al.,^[^ ^32^ ^]^ represent the most extensive GWAS of rsfMRI phenotypes published to date. This investigation explored associations between 1777 intrinsic brain activity phenotypes and 9 026 427 common variants in the UK Biobank (*n* = 47 276). A total of 191 traits were selected for GWASs, which encompassed 75 amplitude traits indicative of regional spontaneous neural activities, 111 pairwise functional connectivities, and 5 global functional connectivities, spanning the DMN, SN, CEN, somatomotor, attention, limbic, and visual networks. Comprehensive details regarding the included rsfMRI phenotypes can be found in the original publication and Table S19 (Supporting Information).

### MD and Other Common Similar Severe Psychiatric Disorders

GWAS summary‐level statistics for MD were derived from Howard et al.,^[^ ^87^ ^]^ who performed a meta‐analysis of data from 807 553 individuals (246 363 cases and 561 190 controls) across the three largest independent GWAS on depression, including studies by Hyde et al.,^[^ ^88^ ^]^ Howard et al.,^[^ ^89^ ^]^ and Wray et al.^[^ ^90^ ^]^ Summary‐level statistics for bipolar disorder were sourced from a study by Mullins et al.,^[^ ^91^ ^]^ which included 41 917 cases and 371 549 controls of European ancestry. For schizophrenia, schizotypal, and delusional disorders, summary statistics were obtained from the FinnGen consortium's R5 release, comprising 10 118 European cases and 208 674 healthy controls, covering eight traits: schizophrenia, schizotypal disorder, persistent delusional disorders, acute and transient psychotic disorders, induced delusional disorder, schizoaffective disorder, and unspecified nonorganic psychotic disorders. For ASD, data were sourced from the PGC, which included 18 382 European cases and 27 969 healthy controls.

### Common Behaviors

An extensive literature review identified several common behaviors that may significantly influence brain imaging phenotypes and the risk of MD, including smoking status, alcohol drinker status, alcohol consumption frequency weekly, and various physical activities:^[^ ^76^, ^77^, ^78^ ^]^ smoking status was defined as whether an individual was a current or former smoker;^[^ ^92^ ^]^ alcohol drinker status was defined based on whether an individual was an alcoholic,^[^ ^93^ ^]^ while alcohol frequency was measured weekly to assess both the frequency and quantity of intake.^[^ ^92^ ^]^ Physical activities over a four‐week period were evaluated, including walking for leisure (excluding transportation), exercises (e.g., swimming, cycling, keep fit, bowling), strenuous sports, and DIY tasks (light tasks like pruning and heavy tasks like weeding and carpentry).^[^ ^93^ ^]^ GWAS summary statistics for these behaviors were obtained from the UK Biobank, with sample sizes detailed in Table S17 (Supporting Information).

### Selection Criteria and Quality Control of Genetic IVs

A rigorous filtering process for the IVs was conducted. First, like previous studies,^[^ ^49^, ^94^, ^95^ ^]^ only SNPs were included that were significantly associated with the 191 human brain functional networks from the rsfMRI datasets at a threshold of *P* < 5 × 10⁻⁸. In addition, to obtain more accurate and objective results for daily behavioral traits, SNPs were also selected based on the stringent threshold of *P* < 5 × 10⁻⁸.^[^ ^23^, ^24^ ^]^ However, for the 83 brain region volumes from human brain morphometry datasets, applying a threshold of *P* < 5 × 10⁻⁸ resulted in too few SNPs being included, which was insufficient for conducting further analyses. Therefore, based on prior research,^[^ ^31^ ^]^ SNPs were selected using a moderately threshold of *P* < 5 × 10⁻⁶.^[^ ^23^, ^24^ ^]^ Second, LD pruning was performed to ensure independence among selected SNPs using the European population from the 1000 Genomes Project as the reference panel. Consistent with previous studies and considering the diminished genetic influence on brain functional networks,^[^ ^49^, ^94^, ^95^ ^]^ SNPs within a clumping window of 1000 kb were pruned for 191 human brain functional networks with an R^2^ < 0.001. In contrast, for other exposures, such as the 83 brain region volumes and common behaviors, the commonly used window size of 10 000 kb was maintained. Third, remaining SNPs were checked using PhenoScanner V2 for strong associations with confounders like education and socioeconomic status.^[^ ^96^ ^]^ To improve the robustness of the genetic instruments, heterogeneity tests were then proposed to detect outliers and adjusted them. Cochran's *Q* test was used for IVW model fitting in the case of horizontal pleiotropy effects, exploiting the “IVW_radial” (alpha = 0.05, weights = 3) function to calculate the modified *Q* and *Q*′ test, respectively, discarding outliers with a nominal significance level of 0.05. Outlier pleiotropic SNPs that could introduce bias using a heterogeneity test (Cochran's *Q* test for IVW model fitting) with the RadialMR method and a *P*‐value < 0.05 were then systematically identified and excluded.^[^ ^97^ ^]^

### Data Harmonization

Before conducting MR analyses, SNPs for both exposure and outcome were harmonized. SNP information associated with the exposure was extracted, and palindromic SNPs (e.g., A/T or G/C alleles) with a minor allele frequency near 0.5, as well as ambiguous and duplicated SNPs, were removed. This harmonization ensured that the IVs were derived from the same DNA strand and were present in both exposure and outcome datasets.^[^ ^33^, ^34^ ^]^

### Testing Instrument Strength and Statistical Power

To assess the extent of weak instrument bias, the F‐statistic was also utilized to measure the strength of IVs. Specifically, an F‐statistic >10 indicates a low risk of weak instruments in the MR analysis. IVs with *F*‐statistics <10 were excluded, as this suggests they were weak IVs. The *F*‐statistic was calculated as: *F* = *R* ^2^(*N*−*k*−1)/*k*(1−*R* ^2^), where *R* ^2^ represents the proportion of exposure variance explained by the genetic variants, *N* represents the sample size, and *k* is the number of instruments. The *R* ^2^ calculation relies on β (genetic effect size from the exposure GWAS data), s.e. (standard error of effect size), and sample sizes (*N*). The formula for computing *R* ^2^ by a single instrumental variant is *R* ^2^ = *β* ^2^/(*β* ^2^ + s.e.^2^ × *N*). The *R* ^2^ of multiple genetic variants is the sum of *R* ^2^ of each genetic variant.^[^ ^33^, ^34^, ^98^ ^]^

### Statistical Analyses—Genetic Correlation Analysis

To enhance the understanding of the relationships between complex traits, genetic correlation analysis was conducted prior to MR analysis. Genetic correlations between identified brain region volumes, functional networks, and MD, as well as other similar severe psychiatric disorders, were estimated using LD score regression.^[^ ^99^ ^]^ European ancestry data from the 1000 Genomes Project served as the LD reference panel, consistent with the predominantly European ancestry of the GWAS samples. Genetic correlation estimates were filtered to include only HapMap3 SNPs due to their reliable imputation status in most studies.^[^ ^99^ ^]^

### Bidirectional and Multivariable MR Analysis

All MR analyses conformed to three fundamental assumptions: (1) genetic variants must be robustly linked to the exposure in bidirectional MR and with at least one of the various exposures in multivariable MR; (2) genetic variants must not be correlated with confounding factors that could bias the causal relationship between exposure and outcome; and (3) genetic variants should affect the outcome directly via exposure rather than through pleiotropy.^[^ ^33^, ^34^ ^]^

Two‐sample bidirectional MR was used to assess potential causal relationships between 83 brain region volumes, 191 brain functional networks, and MD, as well as other similar severe psychiatric disorders. The IVW method was used as the primary analysis, which provides precise and unbiased causal estimates when the IVs meet MR assumptions.^[^ ^33^, ^34^ ^]^ Benjamini–Hochberg FDR correction is a method used for multiple hypothesis testing that aims to control the FDR, and it was applied to adjust the *P*‐values obtained from the MR‐IVW analysis in the forward MR analyses between 83 brain region volumes, 191 brain functional networks, and MD, as well as other similar severe psychiatric disorders, with a threshold set at 0.05.^[^ ^100^ ^]^ Benjamini–Hochberg FDR correction was performed globally (across all 83 brain volumes and 191 functional networks), and the detailed results are presented in Files S2 and S6 (Supporting Information). To enhance the reliability of the results, causality was also estimated using four additional methods: MR‐Egger regression, weighted median estimator (WME), weighted model, and a simple model.^[^ ^33^, ^34^ ^]^ MR‐Egger regression provides robust estimates even if some IVs are invalid, while the WME method allows for horizontal pleiotropy in up to 50% of genetic variants. The weighted model remains consistent with the largest number of efficient SNPs, and the simple model uses an unweighted approach. If fewer than three genetic instruments were available, only the IVW method was used.^[^ ^33^, ^34^ ^]^

Multivariable MR incorporates genetic variants associated with multiple exposures as instruments, enabling to differentiate total causal effects into direct and indirect effects.^[^ ^101^, ^102^ ^]^ Given the interrelationships between brain imaging phenotypes, the direct contributions of individual brain region volumes or functional networks to the risk of MD onset and similar severe psychiatric disorders, were estimated, adjusting for other identified volumes or networks. Strongly associated SNPs were first extracted separately from the identified brain region volumes or functional networks, subsequently removing LD for each set. Next, the genetic instruments from these multiple SNP sets were combined, clustering them by LD (with *R* ^2^ < 0.001 within a 10 000 kb window for brain volumes or *R* ^2^ < 0.001 within a 1000 kb window for functional networks), and overlapping SNPs were eliminated to ensure their independence. SNP effects and standard errors were extracted from the GWAS summary statistics for all exposures and outcomes, and these were harmonized with the outcome data.^[^ ^101^, ^102^ ^]^ To account for both measured and unmeasured pleiotropy, the multivariable MR extension of the IVW method was employed, maintaining the same parameters used in the univariate MR analysis.

### Mediation MR Analysis

Two‐step MR analysis was performed to investigate whether changes in identified unique brain imaging predictive markers mediate the causal effects of common behaviors on the prevention of MD onset.^[^ ^34^, ^101^ ^]^ The first step estimated the causal effects of habits such as smoking status, drinking alcohol, and physical activities over a four‐week period on MD using forward MR (total effect β). The second step assessed the effects of these habits on identified unique brain region volumes and functional networks (direct effect β1). When there was evidence indicating that the behaviors not only influenced the risk of MD but also impacted its onset in association with unique brain region volumes and functional networks, these unique brain imaging markers as mediators. Their causal effects on MD onset were defined as the direct effect (β2), as shown in Tables 1 and 2. The mediation effect (β3) was calculated using the product of β1 and β2 divided by the total effect β, and standard errors were determined using the delta method.^[^ ^34^, ^101^ ^]^ The mediation proportion, representing the mediation effect as a percentage of the total effect, ranges from 0 to 100%.^[^ ^34^, ^101^ ^]^

### MR Sensitivity Analysis

A series of sensitivity analyses was conducted to address potential issues of consistency and pleiotropy in the causal estimates from the IVW method. Cochran's Q statistic was used to assess heterogeneity among genetic variants, with a *P* value < 0.05 indicating significant heterogeneity in SNP effect estimates.^[^ ^33^, ^34^ ^]^ The MR‐Egger intercept test and MR‐PRESSO global test were employed to detect horizontal pleiotropy. The MR‐Egger intercept indicates the mean pleiotropic effect of all instruments, with a value deviating from zero (*P* < 0.05) signifying directional pleiotropy.^[^ ^33^, ^34^, ^103^ ^]^ The MR‐PRESSO global test evaluates overall horizontal pleiotropy, while the MR‐PRESSO outlier test identifies aberrant SNPs contributing to pleiotropy, where a *P*‐value > 0.05 in this test rules out horizontal pleiotropy.^[^ ^33^, ^34^, ^103^ ^]^ Furthermore, since the results of the multivariable MR analysis indicated that three brain regions identified through bidirectional MR are core predictive brain region volumes that play a synergistic role in association with the onset of MD, VIF analysis was conducted to validate the robustness of these results. This analysis aimed to quantify interdependencies among these three core brain region volumes to determine the presence of multicollinearity. VIF values less than 5 and condition number (κ) less than 10 are widely recognized thresholds for indicating the absence of significant multicollinearity, so these thresholds were applied here.^[^ ^35^, ^36^ ^]^

All analyses were performed using R packages: TwoSampleMR (0.6.6), plinkbinr (0.0.0.9), ieugwasr (1.0.1), GenomicSEM (0.0.5), RadialMR (1.1), MRPRESSO (1.0), car (3.1‐3), and MVMR (0.4) in R software (4.4.2). All statistical tests were two‐sided, with a significance threshold of *P* < 0.05. IVW estimates were considered causal associations only if they aligned in direction and significance with at least one other estimate, such as from MR‐Egger regression, and showed no evidence of pleiotropy or heterogeneity. MR estimates were reported as ORs, β coefficients, or proportions, with corresponding 95% CIs. The threshold for genetic correlation analysis was set at *P* < 0.05 to retain datasets with suggestive evidence.

## Conflict of Interest

The authors declare no conflict of interest.

## Author Contributions

M.‐M.X., N.L., and Y.‐W.L. contributed equally to this work. M.M.X., Y.G., and X.Y.C. conceived and designed the study. Y.M.Z. and X.Y.C. supervised the study. N.L. and Y.W.L. participated in the data collection. Y.G., W.M.Y., and Z.Y.Y. performed the data analyses. M.M.X., N.L., and Y.W.L. prepared the tables and figures. M.M.X. and Y.G. wrote the paper. Y.M.Z. and X.Y.C. critically revised the content. All authors contributed to the acquisition and interpretation of data, proofread the manuscript for significant intellectual content, and provided final approval for the version to be published.

## Supporting information



Supporting Information

Supporting Information

Supporting Information

Supporting Information

Supporting Information

Supporting Information

Supporting Information

Supporting Information

Supporting Information

Supporting Information

Supporting Information

Supporting Information

Supporting Information

Supporting Information

Supporting Information

Supporting Information

Supporting Information

Supporting Information

Supporting Information

## Data Availability

The human brain morphometry were sourced from https://onlinelibrary.wiley.com/doi/10.1002/hbm.26283. The human brain functional networks are derived from https://www.nature.com/articles/s41588‐022‐01039‐6. The MD were obtained from the study by https://www.nature.com/articles/s41593‐018‐0326‐7. The GWAS data for bipolar disorder were derived from https://www.nature.com/articles/s41588‐021‐00857‐4. The schizophrenia, schizotypal disorder, and delusional disorders can be accessed at https://gwas.mrcieu.ac.uk/datasets/finn‐b‐F5_SCHIZO/. The ASD can be obtained at https://gwas.mrcieu.ac.uk/datasets/ieu‐a‐1185/. The daily behavioral habits are available from the GWAS catalog with the IDs: GCST90267302, GCST90267266, GCST90042709, GCST90044423, GCST90044425, GCST90044426, GCST90044427, and GCST90044428.
