# Supplementary material for: Genetic Evidence Supporting a Role for Brain Region Volume and Functional Network Alterations in Major Depression
Source: Adv Sci (Weinh). 2025 Jul 11;12(37):e06032. doi: 10.1002/advs.202506032 (PMC12499405; doi:10.1002/advs.202506032)
Supplement: Supplementary file 1 — Supporting Information [file ADVS-12-e06032-s001.docx]

**Supplemental Table 1 | LD score regression analysis and sensitivity analyses of associations between identified brain region volumes and major depression and other common similar severe psychiatric disorders**

| **Exposure** | **Outcome** | **Heterogeneity test *P*-value** | **MR-Egger intercept test** | | **No. of SNP outliers excluded** | **MR-PRESSO Global test *P*-value** | **LD score regression analysis** | | |
| --- | --- | --- | --- | --- | --- | --- | --- | --- | --- |
|  |  |  | **Egger_ intercept** | ***P*-value** |  |  | **rg** | **rg_se** | **rg_*P*-value** |
| Left ventral diencephalon volume | Major depression | 0.28868 | 0.00016 | 0.93606 | 24 | 0.248 | -0.09607 | 0.03070 | 0.00175 |
| Right ventral diencephalon volume |  | 0.43662 | 0.00078 | 0.67045 | 23 | 0.513 | -0.07827 | 0.02977 | 0.00855 |
| Thalamus volume |  | 0.35317 | 0.00029 | 0.88944 | 13 | 0.279 | -0.02037 | 0.02930 | 0.48680 |
| Entorhinal cortex volume | Bipolar disorder | 0.80953 | -0.00643 | 0.50988 | 0 | 0.822 | 0.01999 | 0.04098 | 0.62563 |
| Left superior frontal gyrus volume |  | 0.63331 | -0.00073 | 0.89555 | 14 | 0.737 | 0.04489 | 0.03038 | 0.13950 |
| Posterior cingulate cortex volume |  | 0.76269 | -0.00510 | 0.46383 | 6 | 0.718 | -0.00899 | 0.03868 | 0.81628 |
| Right superior frontal gyrus volume |  | 0.14036 | 0.00098 | 0.87479 | 19 | 0.188 | 0.04033 | 0.03127 | 0.19714 |
| Caudal anterior cingulate cortex volume | Schizophrenia, schizotypal and delusional disorders | 0.62154 | 0.03077 | 0.09830 | 0 | 0.645 | 0.02027 | 0.09323 | 0.82791 |
| Caudal middle frontal gyrus volume |  | 0.34677 | -0.01050 | 0.34527 | 0 | 0.419 | 0.13344 | 0.09085 | 0.14188 |
| Fusiform gyrus volume |  | 0.35023 | 0.00097 | 0.94687 | 0 | 0.266 | 0.06482 | 0.08530 | 0.44727 |
| Middle temporal gyrus volume |  | 0.16078 | 0.00190 | 0.88549 | 0 | 0.127 | 0.05109 | 0.09333 | 0.58414 |

**Supplementary Table 1 (continued) | LD score regression analysis and sensitivity analyses of associations between identified brain region volumes and major depression and other common similar severe psychiatric disorders**

| **Exposure** | **Outcome** | | **Heterogeneity test *P*-value** | | | **MR-Egger intercept test** | | | | **No. of SNP outliers excluded** | | **MR-PRESSO global test *P*-value** | | **LD score regression analysis** | | | | |
| --- | --- | --- | --- | --- | --- | --- | --- | --- | --- | --- | --- | --- | --- | --- | --- | --- | --- | --- |
|  |  |  |  |  |  | **Egger_ intercept** | | ***P*-value** | |  |  |  |  | **rg** | | **rg_se** | | **rg_*P*-value** |
| Insular cortex volume | | Autism spectrum disorder | | 0.26443 | 0.00489 | | 0.51789 | | 0 | | 0.092 | | 0.11138 | | 0.04600 | | 0.01547 | |
| Nucleus accumbens volume | |  |  | 0.65247 | 0.00184 | | 0.82832 | | 6 | | 0.710 | | -0.05234 | | 0.04781 | | 0.27361 | |
| Rostral anterior cingulate cortex volume | |  |  | 0.60486 | 0.00316 | | 0.79496 | | 2 | | 0.568 | | 0.04630 | | 0.06138 | | 0.45063 | |

MR, mendelian randomization; LD, linkage disequilibrium; SNP, single nucleotide polymorphism; MR-PRESSO, mendelian randomization pleiotropy RESidual sum and outlier. The number of SNP outliers identified and excluded using MR-PRESSO, RadialMR and PhenoScanner methods. The analysis of heterogeneity and horizontal pleiotropy primarily assessed the robustness of the MR-Inverse Variance Weighted method's results. To pursue a high level of precision in differential outcomes, this study meticulously retained the data to an accuracy of five decimal places. All statistical tests were two-sided. A *P*-value < 0.05 was considered significant.
