# Supplementary material for: Genetic Evidence Supporting a Role for Brain Region Volume and Functional Network Alterations in Major Depression
Source: Adv Sci (Weinh). 2025 Jul 11;12(37):e06032. doi: 10.1002/advs.202506032 (PMC12499405; doi:10.1002/advs.202506032)
Supplement: Supplementary file 3 — Supporting Information [file ADVS-12-e06032-s019.docx]

**Supplemental Table 3 | Quantification of multicollinearity among identified core predictive brain region volumes associated with MD onset based on** **variance inflation factor** **analysis**

| **Left ventral diencephalon volume as** **response variable** | | | |
| --- | --- | --- | --- |
| **Variable** | **VIF** | **Collinearity level** | **Reference range** |
| Right ventral diencephalon volume | 1.21201 | Negligible | VIF <5 ^[1, 2]^ |
| Thalamus volume | 1.21201 | Negligible |  |
| Condition number (κ) | 1.65 | Ideal | κ <10 ^[2]^ |
| **Right ventral diencephalon volume as response variable** | | | |
| **Variable** | **VIF** | **Collinearity level** | **Reference range** |
| Left ventral diencephalon volume | 1.55428 | Negligible | VIF <5 ^[1, 2]^ |
| Thalamus volume | 1.55428 | Negligible |  |
| Condition number (κ) | 2.09 | Ideal | κ <10 ^[2]^ |
| **Thalamus volume as response variable** | | | |
| **Variable** | **VIF** | **Collinearity level** | **Reference range** |
| Left ventral diencephalon volume | 1.19765 | Negligible | VIF <5 ^[1, 2]^ |
| Right ventral diencephalon volume | 1.19765 | Negligible |  |
| Condition number (κ) | 1.63 | Ideal | κ <10 ^[2]^ |

VIF, variance inflation factor. VIF calculated using Type II sum of squares. The VIF is a statistical measure used to assess the severity of multicollinearity among independent variables in a regression model. A VIF value less than the conservative threshold of 5 indicates that the level of multicollinearity is negligible, suggesting that the variable is independent of the others. The symbol κ represents the condition number, which is used to evaluate the stability of a matrix and the degree of multicollinearity. A larger κ value indicates a stronger correlation between variables, increasing the risk of inflated model estimation errors. If κ is significantly less than the conservative threshold of 10, it suggests that the matrix model has strong stability. To pursue a high level of precision in differential outcomes, this study meticulously retained the data to an accuracy of five decimal places.

**References**

[1] O'Brien, RM. A caution regarding rules of thumb for variance inflation factors. Quality & Quantity. 2007;41: 673-90. 10.1007/s11135-006-9018-6

[2] Kim JH. Multicollinearity and misleading statistical results. Korean J Anesthesiol. 2019;72(6): 558-69. 10.4097/kja.19087
