# Supplementary material for: Genetic Evidence Supporting a Role for Brain Region Volume and Functional Network Alterations in Major Depression
Source: Adv Sci (Weinh). 2025 Jul 11;12(37):e06032. doi: 10.1002/advs.202506032 (PMC12499405; doi:10.1002/advs.202506032)
Supplement: Supplementary file 7 — Supporting Information [file ADVS-12-e06032-s013.docx]

**Supplemental Table 7 | LD score regression analysis and sensitivity analyses of associations between identified brain functional networks and major depression and other common similar severe psychiatric disorders**

| **Exposure** | | | | **Outcome** | **Heterogeneity test *P*-value** | **MR-Egger intercept test** | | **No. of SNP outliers excluded** | **MR-PRESSO Global test *P*-value** | **LD score regression analysis** | | |
| --- | --- | --- | --- | --- | --- | --- | --- | --- | --- | --- | --- | --- |
| **Location** | **rsfMRI network** | | |  |  | **Egger_ intercept** | ***P*-value** |  |  | **rg** | **rg_se** | **rg_**  ***P*-value** |
| edge_pheno1175  (Precuneus\|Cuneus\|Cingulate)&(Frontal) | | (Default_mode\|Central_executive)&(Salience\|Default_mode) | | Major depression | 0.26304 | NA | NA | 0 | NA | -0.19142 | 0.05264 | 0.00028 |
| edge_pheno1269  (Parietal)&(Frontal) | | (Central_executive\|Attention)&(Central_executive\|Salience\|Default_mode) | |  | 0.74323 | NA | NA | 0 | NA | 0.00916 | 0.04524 | 0.83950 |
| edge_pheno1122  (Temporal)&(Frontal_Inf) | | | (Default_mode)&(Default_mode\|Central_executive) | Bipolar disorder | 0.18005 | -0.00539 | 0.72634 | 0 | 0.161 | -0.04453 | 0.03818 | 0.24346 |
| edge_pheno1134  (Frontal_Sup)&(Frontal_Inf) | | | (Default_mode\|Central_executive)&(Default_mode\|Central_executive) |  | 0.93690 | 0.00503 | 0.87167 | 0 | NA | 0.00641 | 0.03249 | 0.84368 |
| edge_pheno1273  (Supp_Motor_Area\|Frontal)&(Frontal) | | | (Salience\|Default_mode)&(Central_executive\|Salience\|Default_mode) |  | 0.42654 | -0.09749 | 0.63396 | 0 | 0.252 | 0.14372 | 0.04541 | 0.00155 |
| edge_pheno1296  (Precuneus\|Angular\|Cingulate)&(Temporal) | | | (Default_mode\|Central_executive)&(Default_mode\|Central_executive) |  | 0.45280 | -0.00395 | 0.87340 | 0 | 0.484 | 0.01110 | 0.04067 | 0.78490 |

**Supplementary Table 7 (continued) | LD score regression analysis and sensitivity analyses of associations between identified brain functional networks and major depression and other common similar severe psychiatric disorders**

| **Exposure** | | | | **Outcome** | | **Heterogeneity test *P*-value** | | **MR-Egger intercept test** | | **No. of SNP outliers excluded** | | **MR-PRESSO Global test *P*-value** | | **LD score regression analysis** | | | | | |
| --- | --- | --- | --- | --- | --- | --- | --- | --- | --- | --- | --- | --- | --- | --- | --- | --- | --- | --- | --- |
| **Location** | **rsfMRI network** | | |  |  |  |  | **Egger_ intercept** | ***P*-value** |  |  |  |  | **rg** | | **rg_se** | | **rg_**  ***P*-value** | |
| edge_pheno1382  (Frontal_Inf)&(Temporal_Mid\|Angular) | | | | (Default_mode\|Central_executive)&(Default_mode) | | Bipolar disorder | | 0.15266 | 0.00292 | 0.94153 | 0 | 0.270 | | -0.00044 | | 0.04232 | | 0.99163 | |
| edge_pheno918  (Precuneus\|Angular\|Cingulate)&(Precuneus\|Parietal_Sup) | | | (Default_mode\|Central_executive)&(Attention\|Central_executive) | | | Schizophrenia, schizotypal and delusional disorders | | 0.18285 | -0.09547 | 0.59483 | 0 | NA | | 0.16918 | | 0.14164 | | 0.23230 | |
| edge_pheno1309  (Parietal)&(Temporal) | | | (Attention\|Central_executive\|Salience)&(Default_mode\|Central_executive) | | |  |  | 0.70544 | -0.02263 | 0.78400 | 0 | 0.823 | | 0.06451 | | 0.10892 | | 0.55367 | |
| node_pheno19  Occipital | | | Visual | | |  |  | 0.80712 | 0.03487 | 0.75919 | 0 | 0.359 | | -0.03786 | | 0.14216 | | 0.78997 | |
| node_pheno31  (Occipital\|Precuneus) | | | (Default_mode\|Central_executive) | | |  |  | 0.35908 | 0.01002 | 0.70223 | 0 | 0.346 | | -0.09912 | | 0.10160 | | 0.32926 | |
| node_pheno35  (Cuneus\|Occipital) | | | Visual | | |  |  | 0.34794 | 0.08875 | 0.11199 | 0 | 0.373 | | -0.00895 | | 0.12951 | | 0.94492 | |

**Supplementary Table 7 (continued) | LD score regression analysis and sensitivity analyses of associations between identified brain functional networks and major depression and other common similar severe psychiatric disorders**

| **Exposure** | | | **Outcome** | | **Heterogeneity test *P*-value** | | | **MR-Egger intercept test** | | | **No. of SNP outliers excluded** | | **MR-PRESSO Global test *P*-value** | | | **LD score regression analysis** | | | | | |
| --- | --- | --- | --- | --- | --- | --- | --- | --- | --- | --- | --- | --- | --- | --- | --- | --- | --- | --- | --- | --- | --- |
| **Location** | **rsfMRI network** | |  |  |  |  |  | **Egger_ intercept** | | ***P*-value** |  |  |  |  |  | **rg** | **rg_se** | | | **rg_**  ***P*-value** | |
| node_pheno37  Occipital | | Visual | | Schizophrenia, schizotypal and delusional disorders | | 0.17020 | 0.05976 | | 0.85505 | | | 0 | | NA | 0.04437 | | | 0.17720 | 0.80228 | |  |
| node_pheno39  Parietal | | (Attention\|Central_executive\|Salience) | |  |  | 0.48176 | 0.01666 | | 0.36822 | | | 0 | | 0.501 | 0.03574 | | | 0.10078 | 0.72286 | |  |
| node_pheno57  Precuneus | | (Default_mode\|Central_executive) | |  |  | 0.38313 | 0.07696 | | 0.05031 | | | 0 | | 0.447 | 0.07526 | | | 0.09680 | 0.43688 | |  |
| node_pheno60  (Precuneus\|Parietal_Sup) | | (Attention\|Central_executive) | |  |  | 0.42874 | 0.04324 | | 0.20221 | | | 0 | | 0.417 | 0.01156 | | | 0.10081 | 0.90868 | |  |
| edge_pheno1134  (Frontal_Sup)&(Frontal_Inf) | | (Default_mode\|Central_executive)&(Default_mode\|Central_executive) | | Autism spectrum disorder | | 0.64176 | 0.02905 | | 0.57497 | | | 0 | | NA | 0.07977 | | | 0.05216 | 0.12617 | |  |

**Supplementary Table 7 (continued) | LD score regression analysis and sensitivity analyses of associations between identified brain functional networks and major depression and other common similar severe psychiatric disorders**

| **Exposure** | | | | | **Outcome** | | **Heterogeneity test *P*-value** | | | **MR-Egger intercept test** | | | | **No. of SNP outliers excluded** | | **MR-PRESSO Global test *P*-value** | | **LD score regression analysis** | | | | |
| --- | --- | --- | --- | --- | --- | --- | --- | --- | --- | --- | --- | --- | --- | --- | --- | --- | --- | --- | --- | --- | --- | --- |
| **Location** | **rsfMRI network** | | | |  |  |  |  |  | **Egger_ intercept** | | ***P*-value** | |  |  |  |  | **rg** | | **rg_se** | | **rg_**  ***P*-value** |
| edge_pheno1184  (Supp_Motor_Area\|Frontal)&(Frontal) | | | | (Salience\|Default_mode)&(Salience\|Default_mode) | | Autism spectrum disorder | | 0.69718 | NA | | NA | | 0 | | NA | | -0.02497 | | 0.07676 | | 0.74492 | |
| node_pheno10  (Postcentral\|Precentral) | | | | (Postcentral\|Precentral-Motor) | |  |  | 0.82912 | 0.05644 | | 0.25426 | | 0 | | 0.655 | | -0.13199 | | 0.06992 | | 0.05907 | |
| node_pheno12  (Paracentral\|Postcentral) | | | Motor | | |  |  | 0.96337 | 0.01102 | | 0.51286 | | 0 | | 0.965 | | -0.07143 | | 0.07033 | | 0.30979 | |
| node_pheno36  (Insula\|Cingulate) | | (Salience\|Default_mode) | | | |  |  | 0.96708 | 0.00351 | | 0.82821 | | 0 | | 0.994 | | -0.04126 | | 0.07011 | | 0.55614 | |
| node_pheno44  Cerebellum | | | (Subcortical-cerebellum) | | |  |  | 0.93131 | 0.01100 | | 0.71521 | | 0 | | 0.932 | | -0.11500 | | 0.05866 | | 0.04993 | |
| node_pheno74  Frontal | | | Limbic | | |  |  | 0.72019 | 0.02096 | | 0.64216 | | 0 | | 0.731 | | -0.07034 | | 0.07976 | | 0.37784 | |

MR, mendelian Randomization; rsfMRI, resting-state functional magnetic resonance images; LD, linkage disequilibrium; SNP, single nucleotide polymorphism; MR-PRESSO, mendelian randomization pleiotropy RESidual sum and outlier; NA, not applicable. NA refers to not enough instrumental variables to perform the MR-PRESSO Global test. The number of SNP outliers identified and excluded in the MR-PRESSO, RadialMR and PhenoScanner metahods. The analysis of heterogeneity and horizontal pleiotropy primarily assessed the robustness of the MR-Inverse Variance Weighted method's results. To pursue a high level of precision in differential outcomes, this study meticulously retained the data to an accuracy of five decimal places. All statistical tests were two-sided. A *P*-value < 0.05 was considered significant.
