# Supplementary material for: Genetic Evidence Supporting a Role for Brain Region Volume and Functional Network Alterations in Major Depression
Source: Adv Sci (Weinh). 2025 Jul 11;12(37):e06032. doi: 10.1002/advs.202506032 (PMC12499405; doi:10.1002/advs.202506032)
Supplement: Supplementary file 11 — Supporting Information [file ADVS-12-e06032-s009.docx]

**Supplemental Table 11 | Multivariable MR results of effect estimate between core brain region volume and common clinical severe psychiatric disorders similar to major depression after adjusting for other identified brain region volumes**

| **Exposure** | **Adjustment of brain region volumes** | **Outcome** | **method** | **Beta** | **SE** | **OR（95% CI）** | ***P*-value** |
| --- | --- | --- | --- | --- | --- | --- | --- |
| Entorhinal cortex volume | Three other identified brain region volumes | Bipolar disorder | MVMR-IVW | 0.00011 | 0.00025 | 1.00011  (0.99962 to 1.00060) | 0.65606 |
| Left superior frontal gyrus volume |  |  | MVMR-IVW | -0.00006 | 0.00007 | 0.99994  (0.99980 to 1.00007) | 0.35349 |
| Posterior cingulate cortex volume |  |  | MVMR-IVW | 0.00002 | 0.00018 | 1.00002  (0.99966 to 1.00039) | 0.89479 |
| Right superior frontal gyrus volume |  |  | MVMR-IVW | 0.00015 | 0.00007 | 1.00015  (1.00001 to 1.00028) | 0.03359 |
| Caudal anterior cingulate cortex volume | Three other identified brain region volumes | Schizophrenia, schizotypal and delusional disorders | MVMR-IVW | -0.00024 | 0.00028 | 0.99976  (0.99921 to 1.00032) | 0.40423 |
| Caudal middle frontal gyrus volume |  |  | MVMR-IVW | 0.00020 | 0.00009 | 1.00020  (1.00003 to 1.00038) | 0.02313 |
| Fusiform gyrus volume |  |  | MVMR-IVW | -0.00001 | 0.00009 | 0.99999  (0.99982 to 1.00016) | 0.94546 |
| Middle temporal gyrus volume |  |  | MVMR-IVW | 0.00014 | 0.00007 | 1.00014  (1.00001 to 1.00028) | 0.03932 |

**Supplementary Table 11 (continued) | Multivariable MR results of effect estimate between core brain region volume and common clinical severe psychiatric disorders similar to major depression after adjusting for other identified brain region volumes**

| **Exposure** | **Adjustment of brain region volumes** | **Outcome** | **method** | **Beta** | **SE** | **OR（95% CI）** | ***P*-value** |
| --- | --- | --- | --- | --- | --- | --- | --- |
| Insular cortex volume | Two other identified brain region volumes | Autism spectrum disorder | MVMR-IVW | 0.00025 | 0.00010 | 1.00024  (1.00005 to 1.00044) | 0.01367 |
| Nucleus accumbens volume |  |  | MVMR-IVW | -0.00172 | 0.00095 | 0.99828  (0.99642 to 1.00014) | 0.07011 |
| Rostral anterior cingulate cortex volume |  |  | MVMR-IVW | -0.00055 | 0.00026 | 0.99945  (0.99893 to 0.99996) | 0.03598 |

MVMR, multivariable mendelian randomization; IVW, inverse variance weighted; SE, standard error; OR, odds ratio; CI, confidence interval. ‘MVMR-IVW’ indicate multivariable MVMR via the IVW method. To pursue a high level of precision in differential outcomes, this study meticulously retained the data to an accuracy of five decimal places. All statistical tests were two-sided. A *P*-value < 0.05 was considered significant association.
