# Supplementary material for: Genetic Evidence Supporting a Role for Brain Region Volume and Functional Network Alterations in Major Depression
Source: Adv Sci (Weinh). 2025 Jul 11;12(37):e06032. doi: 10.1002/advs.202506032 (PMC12499405; doi:10.1002/advs.202506032)
Supplement: Supplementary file 14 — Supporting Information [file ADVS-12-e06032-s002.docx]

**Supplemental Table 14 | Multivariable MR results of effect estimate between core brain functional network and** **common clinical severe psychiatric disorders similar to major depression after adjusting for other identified brain functional networks**

| **Exposure** | | | | **Adjustment of brain functional networks** | | **Outcome** | **method** | **Beta** | **SE** | **OR（95% CI）** | ***P*-value** |
| --- | --- | --- | --- | --- | --- | --- | --- | --- | --- | --- | --- |
| **Location** | **rsfMRI network** | | |  |  |  |  |  |  |  |  |
| edge_pheno1122  (Temporal)&(Frontal_Inf) | | (Default_mode)&(Default_mode\|Central_executive) | | Four other identified brain functional network | | Bipolar disorder | MVMR-IVW | -0.15062 | 0.17813 | 0.86018  (0.60668 to 1.21959) | 0.39780 |
| edge_pheno1134  (Frontal_Sup)&(Frontal_Inf) | | (Default_mode\|Central_executive)&(Default_mode\|Central_executive) | |  |  |  | MVMR-IVW | -0.26255 | 0.16458 | 0.76909  (0.55703 to 1.06187) | 0.11066 |
| edge_pheno1273  (Supp_Motor_Area\|Frontal)&(Frontal) | | (Salience\|Default_mode)&(Central_executive\|Salience\|Default_mode) | |  |  |  | MVMR-IVW | 0.16735 | 0.11403 | 1.18217  (0.94539 to 1.47825) | 0.14223 |
| edge_pheno1296  (Precuneus\|Angular\|Cingulate)&(Temporal) | | (Default_mode\|Central_executive)&(Default_mode\|Central_executive) | |  |  |  | MVMR-IVW | 0.27435 | 0.10396 | 1.31567  (1.07313 to 1.61303) | 0.00832 |
| edge_pheno1382  (Frontal_Inf)&(Temporal_Mid\|Angular) | | (Default_mode\|Central_executive)&(Default_mode) | |  |  |  | MVMR-IVW | 0.08736 | 0.23406 | 1.09129  (0.68977 to 1.72654) | 0.70898 |
| edge_pheno918  (Precuneus\|Angular\|Cingulate)&(Precuneus\|Parietal_Sup) | | | (Default_mode\|Central_executive)&(Attention\|Central_executive) | Eight other identified brain functional network | Schizophrenia, schizotypal and delusional disorders | | MVMR-IVW | 0.35904 | 0.35145 | 1.43195  (0.71907 to 2.85157) | 0.30697 |

**Supplementary Table 14 (continued) | Multivariable MR results of effect estimate between core brain functional network and common clinical severe psychiatric disorders similar to major depression after adjusting for other identified brain functional networks**

| **Exposure** | | | **Adjustment of brain functional networks** | | **Outcome** | **method** | **Beta** | **SE** | **OR（95% CI）** | ***P*-value** |
| --- | --- | --- | --- | --- | --- | --- | --- | --- | --- | --- |
| **Location** | **rsfMRI network** | |  |  |  |  |  |  |  |  |
| edge_pheno1309  (Parietal)&(Temporal) | | (Attention\|Central_executive\|Salience)&(Default_mode\|Central_executive) | Eight other identified brain functional network | Schizophrenia, schizotypal and delusional disorders | | MVMR-IVW | 0.30972 | 0.23513 | 1.36304  (0.85973 to 2.16101) | 0.18777 |
| node_pheno19  Occipital | | Visual |  |  |  | MVMR-IVW | 1.13107 | 1.09199 | 3.09898  (0.36450 to 26.34740) | 0.30030 |
| node_pheno31  (Occipital\|Precuneus) | | (Default_mode\|Central_executive) |  |  |  | MVMR-IVW | 0.80196 | 0.74946 | 2.22990  (0.51325-9.68820) | 0.28460 |
| node_pheno35  (Cuneus\|Occipital) | | Visual |  |  |  | MVMR-IVW | -0.65765 | 0.86138 | 0.51807  (0.09576 to 2.80287) | 0.44517 |
| node_pheno37  Occipital | | Visual |  |  |  | MVMR-IVW | -0.97175 | 1.31636 | 0.37842  (0.02867 to 4.99444) | 0.46039 |

**Supplementary Table 14 (continued) | Multivariable MR results of effect estimate between core brain functional network and common clinical severe psychiatric disorders similar to major depression after adjusting for other identified brain functional networks**

| **Exposure** | | | **Adjustment of brain functional networks** | | | **Outcome** | **method** | **Beta** | **SE** | **OR（95% CI）** | ***P*-value** |
| --- | --- | --- | --- | --- | --- | --- | --- | --- | --- | --- | --- |
| **Location** | **rsfMRI network** | |  |  |  |  |  |  |  |  |  |
| node_pheno39  Parietal | | (Attention\|Central_executive\|Salience) | Eight other identified brain functional network | | Schizophrenia, schizotypal and delusional disorders | | MVMR-IVW | -0.31541 | 0.47696 | 0.72949  (0.28643 to 1.85789) | 0.50843 |
| node_pheno57  Precuneus | | (Default_mode\|Central_executive) |  |  |  |  | MVMR-IVW | 0.32490 | 0.35851 | 1.38389  (0.68538 to 2.79429) | 0.36480 |
| node_pheno60  (Precuneus\|Parietal_Sup) | | (Attention\|Central_executive) |  |  |  |  | MVMR-IVW | -0.61049 | 0.56057 | 0.54308  (0.18101 to 1.62941) | 0.27613 |
| edge_pheno1134  (Frontal_Sup)&(Frontal_Inf) | | (Default_mode\|Central_executive)&(Default_mode\|Central_executive) | Six other identified brain functional network | Autism spectrum disorder | | | MVMR-IVW | -0.14077 | 0.15778 | 0.86869  (0.63762 to 1.18351) | 0.37230 |
| edge_pheno1184  (Supp_Motor_Area\|Frontal)&(Frontal) | | (Salience\|Default_mode)&(Salience\|Default_mode) |  |  |  |  | MVMR-IVW | -0.40670 | 0.16678 | 0.66585  (0.48018 to 0.92329) | 0.01475 |

**Supplementary Table 14 (continued) | Multivariable MR results of effect estimate between core brain functional network and common clinical severe psychiatric disorders similar to major depression after adjusting for other identified brain functional networks**

| **Exposure** | | | | **Adjustment of brain functional networks** | | **Outcome** | **method** | **Beta** | **SE** | **OR（95% CI）** | ***P*-value** |
| --- | --- | --- | --- | --- | --- | --- | --- | --- | --- | --- | --- |
| **Location** | **rsfMRI network** | | |  |  |  |  |  |  |  |  |
| node_pheno10  (Postcentral\|Precentral) | | | Motor | Six other identified brain functional network | Autism spectrum disorder | | MVMR-IVW | 0.07566 | 0.68334 | 1.07860  (0.28261 to 4.11654) | 0.91183 |
| node_pheno12  (Paracentral\|Postcentral) | | | Motor |  |  |  | MVMR-IVW | -0.16011 | 0.52916 | 0.85205  (0.30202 to 2.40376) | 0.76221 |
| node_pheno36  (Insula\|Cingulate) | | (Salience\|Default_mode) | |  |  |  | MVMR-IVW | 0.21647 | 0.37256 | 1.24169  (0.59825 to 2.57716) | 0.56122 |
| node_pheno44  Cerebellum | | | (Subcortical-cerebellum) |  |  |  | MVMR-IVW | -0.15099 | 0.53394 | 0.85985  (0.30194 to 2.44862) | 0.77734 |
| node_pheno74  Frontal | | | Limbic |  |  |  | MVMR-IVW | -0.34833 | 0.32719 | 0.70586  (0.37172 to 1.34039) | 0.28705 |

rsfMRI, resting-state functional magnetic resonance images; MVMR, multivariable mendelian randomization; IVW, inverse variance weighted; SE, standard error; OR, odds ratio; CI, confidence interval. ‘MVMR-IVW’ indicate multivariable MVMR via the IVW method. To pursue a high level of precision in differential outcomes, this study meticulously retained the data to an accuracy of five decimal places. All statistical tests were two-sided. A *P*-value < 0.05 was considered significant association.
