# Supplementary material for: Genetic Evidence Supporting a Role for Brain Region Volume and Functional Network Alterations in Major Depression
Source: Adv Sci (Weinh). 2025 Jul 11;12(37):e06032. doi: 10.1002/advs.202506032 (PMC12499405; doi:10.1002/advs.202506032)
Supplement: Supplementary file 17 — Supporting Information [file ADVS-12-e06032-s005.docx]

**Supplemental Table 17 | Overview of the** **GWAS datasets used in the study**

| **Phenotype** | **Year of publication**  **or release** | **Sample size**  **(overall or case/control)** | **Ancestry** | **Consortium or cohort study** | **PubMed identifier** | **IEU OpenGWAS or GWAS catalog ID** |
| --- | --- | --- | --- | --- | --- | --- |
| Human brain morphometry | 2023 | 36,778 | European | UK Biobank | 36987996^[1]^ | -- |
| Human brain functional networks | 2022 | 47,276 | European | UK Biobank | 35393594^[2]^ | -- |
| Major depression | 2019 | 246,363/561,190 | European | Meta | 30718901^[3]^ | ieu-b-102 |
| Bipolar disorder | 2021 | 41,917/371,549 | European | PGC | 34002096^[4]^ | ieu-b-5110 |
| Schizophrenia, schizotypal and delusional disorders | 2021 | 10,118/208,674 | European | FinnGen | -- | finn-b-F5_SCHIZO |
| Autism spectrum disorder | 2017 | 18,382/27,969 | European | PGC | -- | ieu-a-1185 |
| Smoking status | 2023 | 283,749 | European | UK Biobank | 37106081^[5]^ | GCST90267302 |
| Alcohol frequency weekly | 2023 | 283,162 | European | UK Biobank | 37106081^[5]^ | GCST90267266 |
| Alcohol drinker status | 2021 | 425,126/30,529 | European | UK Biobank | 34737426^[6]^ | GCST90042709 |
| Types of physical activity over a 4-week period: walking for pleasure (not as a means of transport) | 2021 | 323,316/128,522 | European | UK Biobank | 34737426^[6]^ | GCST90044423 |
| Types of physical activity over a 4-week period: other exercises (eg: swimming, cycling, keep fit, bowling) | 2021 | 219,338/234,500 | European | UK Biobank | 34737426^[6]^ | GCST90044425 |
| Types of physical activity over a 4-week period: strenuous sports | 2021 | 46,784/407,054 | European | UK Biobank | 34737426^[6]^ | GCST90044426 |
| **Supplementary Table 17** **(continued) \| Overview of the GWAS datasets used in the study** | | | | | | |
| **Phenotype** | **Year of publication**  **or release** | **Sample size**  **(overall or case/control)** | **Ancestry** | **Consortium or cohort study** | **PubMed identifier** | **IEU OpenGWAS or GWAS catalog ID** |
| Types of physical activity over a 4-week period: light DIY (eg: pruning, watering the lawn) | 2021 | 233,765/220,073 | European | UK Biobank | 34737426^[6]^ | GCST90044427 |
| Types of physical activity over a 4-week period: heavy DIY (eg: weeding, lawn mowing, carpentry, digging) | 2021 | 195,399/258,439 | European | UK Biobank | 34737426^[6]^ | GCST90044428 |

GWAS, genome-wide association study; PubMed, public medical; IEU, institute for epidemiology and health care; PGC, psychiatric genomic consortium; DIY, do it yourself. All genetic association estimates were derived from open GWAS studies that integrated both discovery and replication cohorts.

**References**

[1] Fürtjes AE, Arathimos R, Coleman JRI, Cole JH, Cox SR, Deary IJ, et al. General dimensions of human brain morphometry inferred from genome-wide association data. Hum Brain Mapp. 2023;44(8):3311-23. 10.1002/hbm.26283

[2] Zhao B, Li T, Smith SM, Xiong D, Wang X, Yang Y, et al. Common variants contribute to intrinsic human brain functional networks. Nat Genet. 2022;54(4):508-17. 10.1038/s41588-022-01039-6

[3] Howard DM, Adams MJ, Clarke T-K, Hafferty JD, Gibson J, Shirali M, et al. Genome-wide meta-analysis of depression identifies 102 independent variants and highlights the importance of the prefrontal brain regions. Nat Neurosci. 2019;22(3):343-52. 10.1038/s41593-018-0326-7

[4] Mullins N, Forstner AJ, O'Connell KS, Coombes B, Coleman JRI, Qiao Z, et al. Genome-wide association study of more than 40,000 bipolar disorder cases provides new insights into the underlying biology. Nat Genet. 2021;53(6):817-29. 10.1038/s41588-021-00857-4

[5] Schoeler T, Speed D, Porcu E, Pirastu N, Pingault J-B, Kutalik Z. Participation bias in the UK Biobank distorts genetic associations and downstream analyses. Nat Hum Behav. 2023;7(7):1216-27. 10.1038/s41562-023-01579-9

[6] Jiang L, Zheng Z, Fang H, Yang J. A generalized linear mixed model association tool for biobank-scale data. Nat Genet. 2021;53(11):1616-21. 10.1038/s41588-021-00954-4
