# Supplementary material for: Genetic Evidence Supporting a Role for Brain Region Volume and Functional Network Alterations in Major Depression
Source: Adv Sci (Weinh). 2025 Jul 11;12(37):e06032. doi: 10.1002/advs.202506032 (PMC12499405; doi:10.1002/advs.202506032)
Supplement: Supplementary file 18 — Supporting Information [file ADVS-12-e06032-s017.docx]

**Supplemental Table 18 | Detailed information on the human brain morphometry phenotypes**

| **Accession Id** **of** **ebi** | **Reported Trait** | **efoTraits** |
| --- | --- | --- |
| GCST90085819 | Brain stem volume(UKB data field 26526) | brain stem volume measurement |
| GCST90085820 | Left accumbens area volume(UKB data field 26564) | nucleus accumbens volume |
| GCST90085821 | Left amygdala volume (UKB data field 26563) | amygdala volume |
| GCST90085822 | Left banks of superior temporal sulcus volume(UKBdata field 26789) | banks of the superior temporal sulcus volume measurement |
| GCST90085823 | Left caudal anterior cingulate volume(UKB data field 26790) | caudalanterior cingulate cortex volume measurement |
| GCST90085824 | Left caudal middle frontal volume(UKB data field 26791) | caudal middle frontal gyrus volume measurement |
| GCST90085825 | Left caudate volume(UKB data field 26559) | caudate nucleus volume |
| GCST90085867 | Right cuneus volume(UKB data field 26893) | cuneus cortex volume measurement |
| GCST90085826 | Left cuneus volume(UKB data field 26792) | cuneus cortex volume measurement |
| GCST90085827 | Left ventral diencephalon(DC)volume (UKB data field 26565) | ventral diencephalon volume measurement |
| GCST90085828 | Left entorhinalvolume(UKB data field 26793) | entorhinal cortex volume measurement |
| GCST90085829 | Left frontal pole volume(UKB data field 26819) | frontal pole volume measurement |
| GCST90085830 | Left fusiform volume(UKB data field 26794) | fusiform gyrus volume measurement |
| GCST90085831 | Left hippocampus volume(UKB data field 26562) | hippocampal volume |
| GCST90085832 | Left inferior parietal volume(UKB data field 26795) | inferior parietal cortex volume measurement |
| GCST90085833 | Left inferior temporal volume(UKB data field 26796) | inferior temporal gyrus volume measurement |
| GCST90085834 | Left insula volume(UKB data field 26821) | insular cortex volume measurement |
| GCST90085835 | Left isthmus cingulate volume(UKB data field 26797) | isthmuscingulate cortex volume measurement |
| GCST90085836 | Left lateral occipital volume(UKB data field 26798) | lateral occipital cortex volume measurement |
| GCST90085837 | Left lateral orbitofrontal volume(UKB data field26799) | lateral orbital frontal cortex volume measurement |
| GCST90085838 | Left lingual volume(UKB data field 26800) | lingualgyrus volume measurement |

**Supplementary Table 18 (continued) | Detailed information on the human brain morphometry phenotypes**

| **Accession Id** **of** **ebi** | **Reported Trait** | **efoTraits** |
| --- | --- | --- |
| GCST90085839 | Left medial orbitofrontal volume(UKB data field 26801) | medial orbital frontal cortex volume measurement |
| GCST90085840 | Left middle temporal volume(UKB data field 26802) | middle temporal gyrus volume measurement |
| GCST90085841 | Left pallidum volume(UKB data field 26561) | pallidum volume |
| GCST90085842 | Left paracentral volume(UKB data field 26804) | paracentral lobule volume measurement |
| GCST90085843 | Left parahippocampal volume (UKB data field 26803) | parahippocampal gyrus volume measurement |
| GCST90085844 | Left pars opercularis volume(UKB data field 26805) | pars opercularis volume measurement |
| GCST90085845 | Leftpars orbitalis volume(UKB data field 26806) | pars orbitalis volume measurement |
| GCST90085846 | Left pars triangularis volume(UKB data field 26807) | pars triangularis volumemeasurement |
| GCST90085847 | Left pericalcarine volume(UKB data field 26808) | pericalcarine cortex volume measurement |
| GCST90085848 | Left postcentral volume(UKB data field 26809) | postcentral gyrus volume measurement |
| GCST90085849 | Left posterior cingulate volume (UKB data field 26810) | posterior cingulate cortex volume measurement |
| GCST90085850 | Left precentral volume(UKB data field 26811) | precentral gyrus volume measurement |
| GCST90085851 | Left precuneus volume(UKB data field 26812) | precuneus cortex volume measurement |
| GCST90085852 | Left putamen volume (UKB data field 26560) | putamen volume |
| GCST90085853 | Left rostral anterior cingulate volume(UKB data field 26813) | rostral anterior cingulate cortex volume measurement |
| GCST90085854 | Left rostral middle frontal volume(UKB data field 26814) | rostral middle frontal gyrus volume measurement |
| GCST90085855 | Left superior frontal volume(UKB data field 26815) | superior frontal gyrus volume measurement |
| GCST90085856 | Left superior parietal volume(UKB data field 26816) | superior parietal cortex volume measurement |
| GCST90085857 | Left superior temporal volume(UKB data field 26817) | superior temporal gyrus volume measurement |
| GCST90085858 | Left supramarginal volume(UKB data field 26818) | supramarginal gyrus volume measurement |
| GCST90085859 | Left thalamus proper volume(UKB data field 26558) | thalamus volume |

**Supplementary Table 18 (continued) | Detailed information on the human brain morphometry phenotypes**

| **Accession Id** **of** **ebi** | **Reported Trait** | **efoTraits** |
| --- | --- | --- |
| GCST90085860 | Left transverse temporal volume(UKB data field 26820) | transverse temporal cortex volume measurement |
| GCST90085861 | Right accumbens area volume (UKB data field 26595) | nucleus accumbens volume |
| GCST90085862 | Right amygdala volume (UKB data field 26594) | amygdala volume |
| GCST90085863 | Right banks of superior temporal sulcus volume(UKB data field 26890) | banks of the superior temporal sulcus volume measurement |
| GCST90085864 | Right caudal anterior cingulate volume(UKB data field 26891) | caudalanterior cingulate cortex volume measurement |
| GCST90085865 | Right caudal middle frontal volume(UKB data field 26892) | caudal middle frontal gyrus volume measurement |
| GCST90085866 | Right caudate volume(UKB data field 26590) | caudate nucleus volume |
| GCST90085868 | Right ventral diencephalon(DC)volume(UKB data field 26596) | ventral diencephalon volume measurement |
| GCST90085869 | Right entorhinal volume(UKB data field 26894) | entorhinal cortex volume measurement |
| GCST90085870 | Right frontal pole volume(UKB data field 26920) | frontal pole volume measurement |
| GCST90085871 | Right fusiform volume(UKB data field 26895) | fusiform gyrus volume measurement |
| GCST90085872 | Right hippocampus volume(UKB data field 26593) | hippocampal volume |
| GCST90085873 | Right inferior parietal volume(UKB data field 26896) | inferior parietal cortex volume measurement |
| GCST90085874 | Right inferior temporal volume(UKB data field 26897) | inferior temporal gyrus volume measurement |
| GCST90085875 | Right insula volume(UKB data field 26922) | insular cortex volume measurement |
| GCST90085876 | Right isthmus cingulate volume(UKB data field 26898) | isthmus cingulate cortex volume measurement |
| GCST90085877 | Right lateral occipital volume(UKB data field 26899) | lateral occipital cortex volume measurement |
| GCST90085878 | Right lateral orbitofrontal volume(UKB data field 26900) | lateral orbital frontal cortex volume measurement |
| GCST90085879 | Right lingual volume(UKB data field 26901) | lingual gyrus volume measurement |
| GCST90085880 | Right medial orbitofrontal volume(UKB data field 26902) | medial orbital frontal cortex volume measurement |
| GCST90085881 | Right middle temporal volume(UKB data field 26903) | middle temporalgyrus volume measurement |

**Supplementary Table 18 (continued) | Detailed information on the human brain morphometry phenotypes**

| **Accession Id** **of** **ebi** | **Reported Trait** | **efoTraits** |
| --- | --- | --- |
| GCST90085882 | Right pallidum volume(UKB data field 26592) | palldum volume |
| GCST90085883 | Right paracentral volume(UKB data field 26905) | paracentral lobule volume measurement |
| GCST90085884 | Right parahippocampal volume(UKB data field 26904) | parahippocampal gyrus volume measurement |
| GCST90085885 | Right pars opercularis volume (UKB data field 26906) | pars opercularis volume measurement |
| GCST90085886 | Right pars orbitalis volume (UKB data field 26907) | pars orbitalis volume measurement |
| GCST90085887 | Right pars tiangularis volume(UKB data field 26908) | parstiangularis volume measurement |
| GCST90085888 | Right pericalcarinevolume(UKB data field 26909) | pericalcarine cortex volume measurement |
| GCST90085889 | Right postcentral volume(UKB data field 26910) | postcentral gyrus volume measurement |
| GCST90085890 | Right posterior cingulate volume(UKB data field 26911) | posterior cingulate cortex volume measurement |
| GCST90085891 | Right precentral volume(UKB data field 26912) | precentral gyrus volume measurement |
| GCST90085892 | Right precuneus volume(UKB data field 26913) | precuneus cortex volume measurement |
| GCST90085893 | Right putamen volume (UKB data field 26591) | putamen volume |
| GCST90085894 | Right rostral anterior cingulate volume(UKB data field 26914) | rostral anterior cingulate cortex volume measurement |
| GCST90085895 | Right rostral middle frontal volume(UKB data field 26915) | rostralmiddlefrontal gyrus volume measurement |
| GCST90085896 | Right superior frontal volume (UKB data field 26916) | superior frontalgyrus volumemeasurement |
| GCST90085897 | Right superior parietal volume(UKB data field 26917) | superior parietal cortex volume measurement |
| GCST90085898 | Right superior temporal volume(UKB data field 26918) | superior temporal gyrus volume measurement |
| GCST90085899 | Right supramarginal volume(UKB data field 26919) | supramarginal gyrus volume measurement |
| GCST90085900 | Rightthalamus proper volume(UKB data field 26589) | thalamus volume |
| GCST90085901 | Right transverse temporal volume(UKB data field 26921) | transverse temporal cortex volume measurement |

Fürtjes AE, Arathimos R, Coleman JRI, Cole JH, Cox SR, Deary IJ, et al. General dimensions of human brain morphometry inferred from genome-wide association data. Hum Brain Mapp. 2023;44(8):3311-23. 10.1002/hbm.26283
